# Paeoniflorin and Hydroxysafflor Yellow A in Xuebijing Injection Attenuate Sepsis-Induced Cardiac Dysfunction and Inhibit Proinflammatory Cytokine Production

**DOI:** 10.3389/fphar.2020.614024

**Published:** 2021-04-13

**Authors:** Xin-Tong Wang, Zhen Peng, Ying-Ying An, Ting Shang, Guangxu Xiao, Shuang He, Xi Chen, Han Zhang, Yuefei Wang, Tao Wang, Jun-Hua Zhang, Xiumei Gao, Yan Zhu, Yuxin Feng

**Affiliations:** ^1^ State Key Laboratory of Component-based Chinese Medicine, Tianjin University of Traditional Chinese Medicine, Tianjin, China; ^2^ Research and Development Center of TCM, Tianjin International Joint Academy of Biotechnology and Medicine, Tianjin, China

**Keywords:** Xuebijing injection, septic shock, sepsis-induced myocardial dysfunction, paeoniflorin (Pae), hydroxysafflor yellow A (HSYA), cytokine storm, CXCL2/MIP-2

## Abstract

Sepsis-induced myocardial dysfunction is a major contributor to the poor outcomes of septic shock. As an add-on with conventional sepsis management for over 15 years, the effect of Xuebijing injection (XBJ) on the sepsis-induced myocardial dysfunction was not well understood. The material basis of Xuebijing injection (XBJ) in managing infections and infection-related complications remains to be defined. A murine cecal ligation and puncture (CLP) model and cardiomyocytes *in vitro* culture were adopted to study the influence of XBJ on infection-induced cardiac dysfunction. XBJ significantly improved the survival of septic-mice and rescued cardiac dysfunction *in vivo*. RNA-seq revealed XBJ attenuated the expression of proinflammatory cytokines and related signalings in the heart which was further confirmed on the mRNA and protein levels. Xuebijing also protected cardiomyocytes from LPS-induced mitochondrial calcium ion overload and reduced the LPS-induced ROS production in cardiomyocytes. The therapeutic effect of XBJ was mediated by the combination of paeoniflorin and hydroxysafflor yellow A (HSYA) (C0127-2). C0127-2 improved the survival of septic mice, protected their cardiac function and cardiomyocytes while balancing gene expression in cytokine-storm-related signalings, such as TNF-α and NF-κB. In summary, Paeoniflorin and HSYA are key active compounds in XBJ for managing sepsis, protecting cardiac function, and controlling inflammation in the cardiac tissue partially by limiting the production of IL-6, IL-1β, and CXCL2.

## Highlights

• Xuebijing injection protected cardiac function during systemic infection partially by down-regulating the expression of pro-inflammatory cytokines and related pathways in the cardiac tissue.
• Paeoniflorin and hydroxysafflor yellow A in XBJ rescued mice from septic shock.
• Paeoniflorin and hydroxysafflor yellow A in XBJ protected cardiac function in septic mice partially by inhibiting the expression of pro-inflammatory cytokines/chemokines and related pathways in the cardiac tissue.

**Figure:**
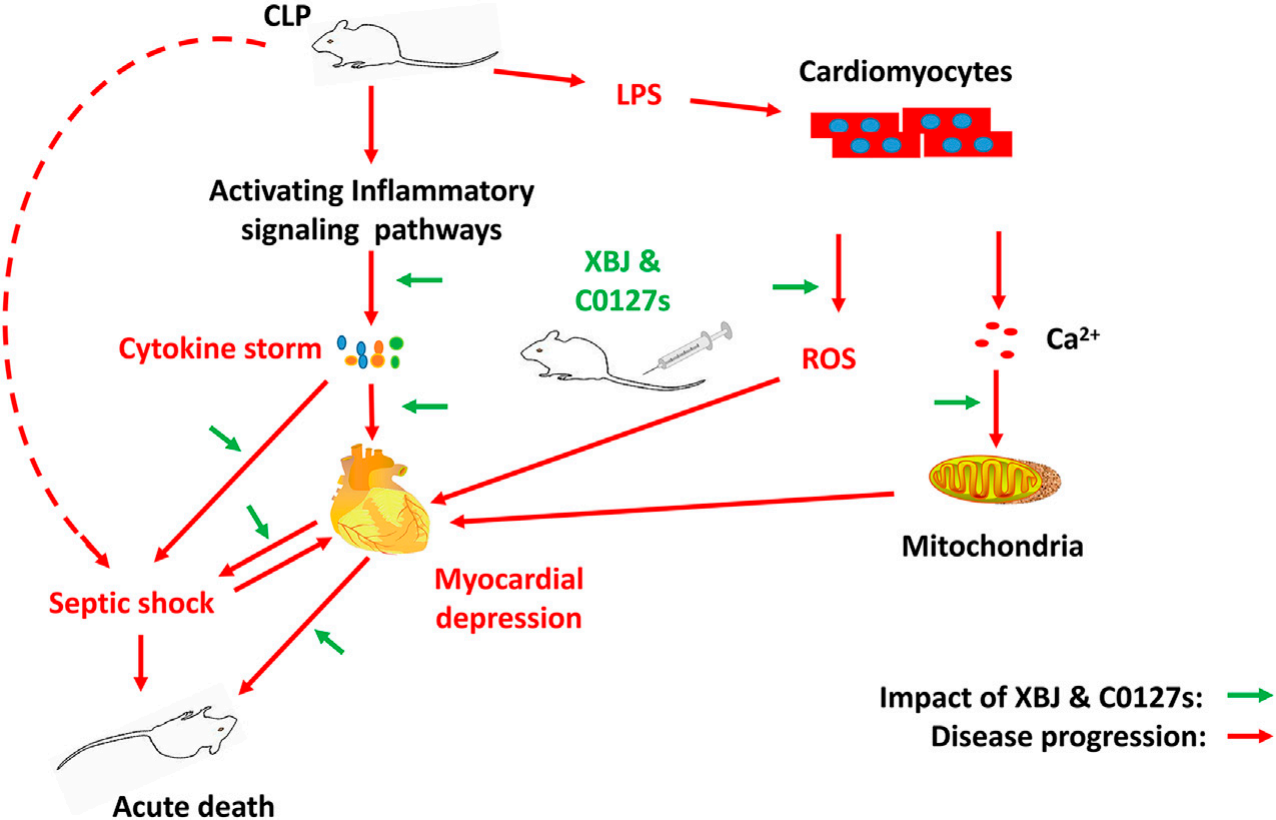
Graphical Abstract |

## Introduction

The mortality rate in septic shock generally exceeds 40% (Beesley et al., 2018). Sepsis-induced cardiomyopathy (SIC), a frequent incident in sepsis, contributes to the poor outcomes of septic shock (Ehrman et al., 2018; Martin et al., 2019). Circulating proinflammatory cytokines act directly on cardiomyocytes and vasculatures to compromise myocardial performance (Ehrman et al., 2018). It is believed that TNF-α, IL-1β, and IL-6 are major contributors to SIC (Pathan et al., 2004; Liu et al., 2017; Martin et al., 2019).

Results of clinical trials suggested that beta-adrenergic agonists and levosimendan were not effective in improving outcomes in sepsis patients (Gordon et al., 2016; Walley, 2018). Medications improving cardiovascular function and targeting proinflammatory cytokines showed therapeutic potentials to manage sepsis and sepsis-induced myocardial dysfunction (Li et al., 2007; Walley, 2018). However, targeting TLR4 signaling with Eritoran did not improve survival in sepsis patients (Opal et al., 2013; Martin et al., 2019). Murine monoclonal anti-TNF-α improved ventricular function in patients with septic shock. But it did not improve patient survival (Vincent et al., 1992; Abraham et al., 1998).

Approved to treat sepsis and septic shock as an add-on in China since 2004, Xuebijing injection significantly improved the survival of severe pneumonia patients in a large-scale clinical trial (Cheng et al., 2016; Zhang et al., 2018; Song et al., 2019). Showing promising clinical effect in managing severe COVID-19 and saving thousands of lives in China, it was recently approved to treat moderate and severe CVOID-19 in China (Fan et al., 2020). Activating circulation and remove stasis, XBJ maintains the balance of the immune system and renders organ protections during systemic infection (Liu et al., 2014a; Chen et al., 2018; Shang et al., 2019). Paeoniflorin claims the highest concentration and hydroxysafflor yellow A claims the 2^nd^ highest concentration among all detectable compounds in Xuebijing injection (Cheng et al., 2016; Shang et al., 2019; Li et al., 2020a). A series of studies indicated these two compounds are promising Xuebijing constituents of therapeutic importance (Cao et al., 2011; Zhai and Guo, 2016b; Cheng et al., 2016; Zou et al., 2018; Shang et al., 2019). Paeoniflorin improved survival and cardiac function in LPS-treated rodents (Cao et al., 2011; Zhai and Guo, 2016b; Liu et al., 2016). HSYA improved ischaemia-induced cardiac haemodynamics by stimulating nucleolin-mediated angiogenesis (Zou et al., 2018).

Though XBJ has been used to treat sepsis as an add-on for over one and a half decades, several key questions remain to be addressed: 1. What’s the influence of XBJ on sepsis-induced cardiac dysfunction? 2. What are the mechanisms of XBJ in managing sepsis-induced cardiac dysfunction? 3. Which compounds in XBJ play major roles in managing septic shock? 4. Which compounds in XBJ play a major role in managing sepsis-induced cardiac dysfunction? In this study, we try to address these questions by evaluating the impact of XBJ and its key compounds (paeoniflorin and hydroxysafflor yellow A) on cardiac function in a clinical relevant shock model (Barth et al., 2006) and investigating the molecular mechanism.

## Materials and Methods

### Chemicals and Reagents

Xuebijing injection (catalog number: z20040033, batch number: 1905061) was manufactured by Tianjin Chase Sun Pharmaceutical Co., Ltd (Tianjin, China). Paeoniflorin (CAS #:23180-57-6), Hydroxysafflor yellow A (CAS #: 78281-02-4), Ferulic acid (CAS # 537- 98-4), and protocatechuic aldehyde (CAS #: 139-85-5) were purchased from Shanghai Yuanye Biotechnology Co., Ltd. (Shanghai, China). Mouse NT-proBNP ELISA (Cat#: ZC-37812-96T) kit, DCFH-DA (Cat#:CA1410-100T), RIPA tissue/cell lysate (Cat#: R0020), SDS-PAGE gel kit, and Rainbow 245 Spectrum Marker (Cat#:PR 1920), Mouse IL-6 ELISA kit (Cat#:ZC-37988-96T), Mouse IL-1β ELISA kit (Cat#:ZC-37974-96T), and EasySee Western Blot Kit (Lot#:O30807) were purchased from Solarbio (Beijing, China). The primary antibody of rabbit anti-CXCL2 (bs-1162R) was purchased from Bioss Inc., (Beijing, China) and GAPDH, 1:4,000 (14C10) was from Cell Signaling Technology (Beverly, MA, United States). The secondary antibody Goat anti-Rabbit IgG (1:4,000) (ZB-2301) was purchased from ZS bio (Beijing, China). 2, 2, 2-Tribromoalcohol (Cat#:T48402) and Bacterial lipopolysaccharide (Cat#: L2880-10 MG) were purchased from Sigma Aldrich (St. Louis, MO, United States). Rhod2-AM (Cat#: zy0129) and Anti-GRP78 (BiP) antibody (ab21685) were purchased from Abcam (Cambridge, MA, United States).

### Animals

This study was carried out following the recommendations of the Guide for the Care and Use of Laboratory Animals (NIH Publication No. 85-23, revised 1996, United States) and the recommendations in the Guidance for the Care and Use of Laboratory Animals issued by the Ministry of Science and Technology of China. All experiments were approved by the Experimental Animal Ethics Committee of Tianjin University of Traditional Chinese Medicine (Tianjin, China) and performed in accordance with its guidelines (license number: TCM-LAE-20170017). Male ICR mice, 6-week-old, were purchased from Beijing Vital River Laboratory Animal Technology Co., Ltd. (Beijing, China, Certificate no.: SCXK Jing 2012-0001). They were kept under controlled temperature (22 ± 2°C) and relative humidity (40% ± 5%) conditions, fed commercial rat food and purified water, and had a 12-h light/dark cycle.

### Cecal Ligation and Puncture

As described previously (Rittirsch et al., 2009; Chen et al., 2018), the abdomen of an anesthetized mouse was depilated, and an incision of less than 1 cm was cut with scissors to expose the cecum which was ligated with a 2–0 line 1/3 from the ileocecal valve. An 18G-needle was used to perforate the cecum (one hole), and then a small amount of feces a little feces was squeezed out. After the operation, the cecum was returned to the original position, the wound was sutured with a 4–0 suture, and the mouse was kept on an electric blanket after the operation.

### Drug Administration

The animals were randomly divided into five groups, sham group, CLP group, CLP + Xuebijing group, CLP + C0127 group, and CLP + C0127-2 group. C0127 was defined as the combination of paeoniflorin (2 mg/ml, CAS #:23180-57-6), hydroxysafflor yellow A (0.5 mg/ml, CAS #: 78281-02-4), ferulic acid (0.02 mg/ml, CAS # 537-98-4), and protocatechuic aldehyde (0.01 mg/ml, CAS #: 139-85-5) at the indicated concentrations (Shang et al., 2019). C0127-2 was defined as the combination of paeoniflorin (2 mg/ml, CAS #:23180-57-6) and hydroxysafflor yellow A (0.5 mg/ml, CAS #: 78281-02-4) at the indicated concentration. Paeoniflorin (2 mg/ml, CAS #:23180-57-6), hydroxysafflor yellow A (0.5 mg/ml, CAS #: 78281-02-4), ferulic acid (0.02 mg/ml, CAS # 537-98-4), and protocatechuic aldehyde (0.01 mg/ml, CAS #: 139-85-5) were dissolved in PBS before the tail-vein injection. Drug treatment was started three days before the cecal ligation and puncture, Xuebijing (9 ml/kg), C0127 (9 ml/kg), C0127-2 (9 ml/kg) were administered twice/day by tail-vein injections to the corresponding groups. Two hrs after CLP, XBJ (9 ml/kg) and C0127s (9 ml/kg) were administered every 12 h by tail-vein injection. Saline was administered to sham and CLP group by every 12 h by tail-vein injection. Mice were feed and supplied with water daily.

### RNA Samples Collection

Twenty-four hours after CLP surgery, the hearts of mice (including normal mice, CLP group, XBJ treatment group, C0127 treatment group, C0127-2 treatment group) were collected. The intracavity blood was washed with normal saline, and the hearts were immediately placed into liquid nitrogen. The hearts were later used for high-throughput sequencing on an Illumina sequencing platform (4 in each group). RNA was extracted from cardiac tissue using standard extraction method as described (Lyu et al., 2018a) and was reverse transcribed using NEB Next®Ultra™RNA library preparation kit for Illumina®.

### RNA-Seq

#### RNA Quantification and Quality Testing

RNA degradation and contamination was monitored on 1% agarose gels. RNA purity was checked using the NanoPhotometer® spectrophotometer (IMPLEN, CA, United States). RNA integrity was assessed using the RNA Nano 6000 Assay Kit of the Bioanalyzer 2100 system (Agilent Technologies, CA, United States).

#### Library Preparation for Transcriptome Sequencing

A total amount of 1 μg RNA per mouse was used as input material for the RNA preparations. Sequencing libraries were generated using NEB Next® UltraTMRNA Library Prep Kit for Illumina® (NEB, United States) following manufacturer’s recommendations and index codes were added to attribute sequences to each sample. Briefly, mRNA was purified from total RNA using poly-T oligo-attached magnetic beads. Fragmentation was carried out using divalent cations under elevated temperature in NEB Next First Strand Synthesis Reaction Buffer (5X). First-strand cDNA was synthesized using random hexamer primer and M-MuLV Reverse transcriptase (RNase H-). Second strand cDNA synthesis was subsequently performed using DNA Polymerase I and RNase H. Remaining overhangs were converted into blunt ends via exonuclease/polymerase activities. After adenylation of 3′ ends of DNA fragments, NEB Next Adaptors with hairpin loop structure were ligated to prepare for hybridization. In order to select cDNA fragments of preferentially 250∼300 bp in length, the library fragments were purified with the AMPureXP system (Beckman Coulter, Beverly, United States). Then 3 μl USER Enzyme (NEB, United States) was used with size-selected, adaptor-ligated cDNA at 37°C for 15 min followed by 5 min at 95°C before PCR. Then PCR was performed with Phusion High-Fidelity DNA polymerase, Universal PCR primers and Index (X) Primer. At last, PCR products were purified (AMPure XP system) and library quality was assessed on the Agilent Bioanalyzer 2100 system.

#### Clustering and Sequencing

The clustering of the index-coded samples was performed on a cBot Cluster Generation System using TruSeq PE Cluster Kit v3-cBot-HS (Illumina) according to the manufacturer’s instructions. After cluster generation, the library preparations were sequenced on an Illumina Novaseq platform and 150 bp paired-end reads were generated.

### Data Analysis

#### Quality Control

Raw data (raw reads) of FastQ format were firstly processed through in-house Perl scripts. In this step, clean data (clean reads) were obtained by removing reads containing adapter, reads containing ploy-N and low quality reads from raw data. At the same time, Q20, Q30 and GC content the clean data were calculated. All the downstream analyses were based on the clean data with high quality.

#### Reads Mapping to the Reference Genome

Reference genome and gene model annotation files were downloaded from genome website directly. Index of the reference genome was built using Hisat2 v2.0.5 and paired-end clean reads were aligned to the reference genome using Hisat2 v2.0.5. Hisat2 was selected as the mapping tool since it can generate a database of splice junctions based on the gene model annotation file and thus a better mapping result than other non-splice mapping tools.

#### Quantification of Gene Expression Levels

Feature Counts v1.5.0-p3 was used to count the reads numbers mapped to each gene. And then FPKM of each gene was calculated based on the length of the gene and reads count mapped to this gene. FPKM, expected number of Fragments Per Kilobase of transcript sequence per Million base pairs sequenced, was used to assess the effect of sequencing depth and gene length for the reads count. It is currently the most commonly used method for estimating gene expression levels.

#### Differential Expression Analysis

For DESeq2 with biological replicates, differential expression analysis of two conditions/groups (two biological replicates per condition) was performed using DESeq2 R package (1.16.1). DESeq2 provides statistical routines for determining differential expression in digital gene expression data using a model based on the negative binomial distribution. The resulting *p*-values were adjusted using the Benjamini and Hochberg’s approach for controlling the false discovery rate. Genes with an adjusted *p*-value <0.05 found by DESeq2 were assigned as differentially expressed. The *p* values were adjusted using the Benjamini and Hochberg method. A corrected *p*-value of 0.05 and absolute fold change of two were set as the threshold for significantly differential expression.

#### GO and KEGG Enrichment Analysis of Differentially Expressed Genes

Gene Ontology (GO) enrichment analysis of differentially expressed genes were implemented using the clusterProfiler R package, in which gene length bias were corrected. GO terms with corrected *p* value less than 0.05 were considered significantly enriched by differential expressed genes. KEGG is a database resource for understanding high-level functions and utilities of the biological system, such as the cell, the organism and the ecosystem, from molecular-level information, especially large-scale molecular datasets generated by genome sequencing and other high-through put experimental technologies (http://www.genome.jp/kegg/). We used clusterProfiler R package to test the statistical enrichment of differential expression genes in KEGG pathways.

### Elisa

Twenty-four hours after CLP, blood was collected and was left at room temperature for 30 min. The clot was removed by centrifuging at 1,500 g for 10 min at 4°C in a centrifuge. The IL-1β, IL-6, and NT-proBNP were detected using ELISA kits by an automatic biochemical analyzer (Multiskan MK3; Thermo Fisher Scientific, Waltham, MA, United States), according to the manufacturer’s instructions as previously described (Lyu et al., 2018a; Chen et al., 2018).

The concentrations of IL-1β and IL-6 in the heart tissue were determined following the manufacturers’ protocol. In brief, the hearts of mice were harvested and rinsed with normal saline 24 h after the CLP procedure. 10ul high-efficiency RIPA lysis buffer for every 1 mg of heart tissue and 10 ul PMSF protease inhibitor for every 1 ml lysis buffer were added to the heart tissue. Later, the tissues was homogenized with an Ultrasonic Tissue Homogenizer (Ningbo Scientz Biotechnology, Ningbo, China) on ice and the tissue supernatant was centrifuged at 13000 g for 5 min. The levels of IL-6 and IL-1β in the cardiac tissue were measured following the manufacturer’s instructions.

### Transthoracic Echocardiography in Mice

Cardiac left ventricular function and coronary blood flow were assessed non-invasively at 24 h after CLP using an ultrahigh resolution small animal ultrasound Vevo 2,100 Imaging System (Visual Sonics, Toronto, ON, Canada) equipped with a 30 MHz transducer (Respress and Wehrens, 2010). As described previously (Respress and Wehrens, 2010), mice were anesthetized (2% isoflurane mixed with 0.5 L/min of 100% O_2_) before imaging. The animals were removed from the induction chamber and the hair on the chest was removed with a depilatory cream. The anesthetized mice were lying on a heating pad with embedded ECG leads to maintain body temperature. Nose cone connected to the anesthesia system was used to maintain a stable sedation level throughout the process (1.0–1.5% isoflurane mixed with 0.5 L/min of 100% O_2_). The level of anesthesia was adjusted to achieve a target heart rate (bpm) of 450 ± 50 beats per minute. Four claws were attached to the ECG electrode with electrode gel. The probe was gently placed on the mouse's chest to locate the left ventricle during the testing. Three cardiac cycles were measured for each mouse and the average value was taken. All data were analyzed after the experiment using the software provided with the ultrasound system.

### Cell Cultures

H9C2 cells were trypsinized, washed, and then seeded in a 96-well black plate at a density of 10,000 cells per well in DMEM medium containing 10% fetal bovine serum. The cells were cultured in an incubator with 37°C, 5% CO_2_ for 24 h. After stimulation with LPS (1ug/ml), Xuebijing (1:20/1:100), C0127 (1:20/1:100), and C0127-2 (1:20/1:100) were added to the cell culture. DCFH-DA (Jiang et al., 2018) and RHOD2 (Joseph et al., 2017) probes were loaded 12 h later. After incubating at 37°C for 30 min and washing 3 times with PBS, images were taken and analyzed with a PerkinElmer high-content imaging system as described (Lyu et al., 2018b).

### Real-Time PCR

Real-time PCR experiments were conducted as described (Xiao et al., 2019). Twenty-four hours after surgery, the mouse hearts were taken out and stored in liquid nitrogen, and then liquid nitrogen was added to pre-cool the mortar. One ml of lysate was added to each tissue sample, and the tissue was ground. The homogenate was separated and the supernatant was removed. RNA was isolated with chloroform, precipitated with isopropyl alcohol, washed with 75% ethanol and was dissolved with ultrapure water. The RNA concentration, purity and integrity were separately measured using Qubit RNA Assay Kit in Qubit 2.0 Fluorometer (Life Technologies, CA, United States), the NanoPhotometer spectrophotometer. Immediately after RNA extraction, total RNA was reverse transcribed into cDNA following the instructions of the Transcriptor First Strand cDNA Synthesis Kit (Roche, Mannheim, Germany). Later real-time PCR was performed in 25ul reaction system. Samples were denatured at 95°C for 30s, annealed at 60°C for 30s, and extended at 72°C for 40s.The relative mRNA level was determined using the comparative CT method and was normalized to the housekeeping gene glyceraldehyde-3-phosphate dehydrogenase (GAPDH). The primers were synthesized by Sangon Company (Shanghai, China).

The primer sequences for real-time PCR were presented in Table 1.

**TABLE 1:** The primer sequences for real-time PCR experiments.

Gene	Forward Primer (5′-3′)	Reverse primer (5′-3′)
GAPDH	TGGTGAAGCAGGCATCTGAG	TGCTGTTGAAGTCGCAGGAG
TLR4	ATGGCATGGCTTACACCACC	GAGGCCAATTTTGTCTCCACA
HMGB1	GGCGAGCATCCTGGCTTATC	GGCTGCTTGTCATCTGCTG
POSTN	CCTGCCCTTATATGCTCTGCT	AAACATGGTCAATAGGCATCACT
GRP78	GTTTGCTGAGGAAGACAAAAAGCTC	CACTTCCATAGAGTTTGCTGATAATTG
IL-6	TAGTCCTTCCTACCCCAATTTCC	TTGGTCCTTAGCCACTCCTTC
IL-1β	GCAACTGTTCCTGAACTCAACT	ATCTTTTGGGGTCCGTCAACT
BNP	CTGAAGGTGCTGTCCCAGATG	GACGGATCCGATCCGGTC
BIRC3	ACGCAGCAATCGTGCATTTTG	CCTATAACGAGGTCACTGACGG
CEBPB	ACACGTGTAACTGTCAGCCG	GCTCGAAACGGAAAAGGTTC
PTGS2	TTCAACACACTCTATCACTGGC	AGAAGCGTTTGCGGTACTCAT
CXCL5	TCCAGCTCGCCATTCATGC	TTGCGGCTATGACTGAGGAAG
CD14	CTCTGTCCTTAAAGCGGCTTAC	GTTGCGGAGGTTCAAGATGTT
FOS	GGTGAAGACCGTGTCAGGAGGCAG	GCCATCTTATTCCGTTCCCTTCGG
CXCL2	CCAACCACCAGGCTACAGG	GCGTCACACTCAAGCTCTG
CXCL12	CGCCAAGG-TCGTCGCCG	TTGGCTCTGGCGATGTGGC
ICAM1	GTGATGCTCAGGTATCCATCCA	CACAGTTCTCAAAGCACAGCG
IL1A	CAGTTCTGCCATTGACCATC	TCTCACTGAAACTCAGCCGT

### Hematoxylin and Eosin Staining

Hematoxylin and eosin (H & E) staining was described previously (Xiao et al., 2019). Briefly, heart tissue was collected 24 h after CLP and was fixed at room temperature in 4% formalin for at least 48 h, dehydrated, and paraffin-embedded. Then the tissue was sectioned in 5-μm thickness, then, stained with H and E at room temperature for 1-2 min. The pictures were taken with an Olympus microscope.

### Western Blot

Western Blot was conducted as described with modifications (Zhai and Guo, 2016a). Firstly, the total protein of heart tissue was extracted with a commercial available Protein Extraction Kit Solarbio (Beijing, China). The protein concentration was determined using the Bio-Rad DC Protein Determination Kit, with bovine serum albumin (BSA) as the standard. The proteins were separated by SDS-PAGE on 12% separation gels, transferred to hydrophobic polyvinylidene (PVDF) membranes, and blocked with TBST (Tris-buffered saline with Tween-20) containing 5% non-fat dry milk. Primary antibodies of CXCL2 (1:1,000 dilution), GAPDH (1:4,000 dilution) were incubated at 4°C overnight. Then, membranes were washed three times with TBST and incubated with secondary antibodies for 2 h at room temperature. The immunoblots were developed using the ECL kit.

### Statistical Analysis

All tests were performed using GraphPad Prism seven software (GraphPad Software, Inc., La Jolla,CA, United States). All experiments were expressed as the mean ± SEM or mean ± SD. Statistical analysis was carried out using Student’s two-tailed t-test for comparison between two groups and One-way analysis of variance (ANOVA) followed by Dunnett’s test the data involved three or more groups. *p* < 0.05 was considered as statistically significant.

## Results

### XBJ Protected the Cardiac Function in a Septic Shock Model

**FIGURE 1:**
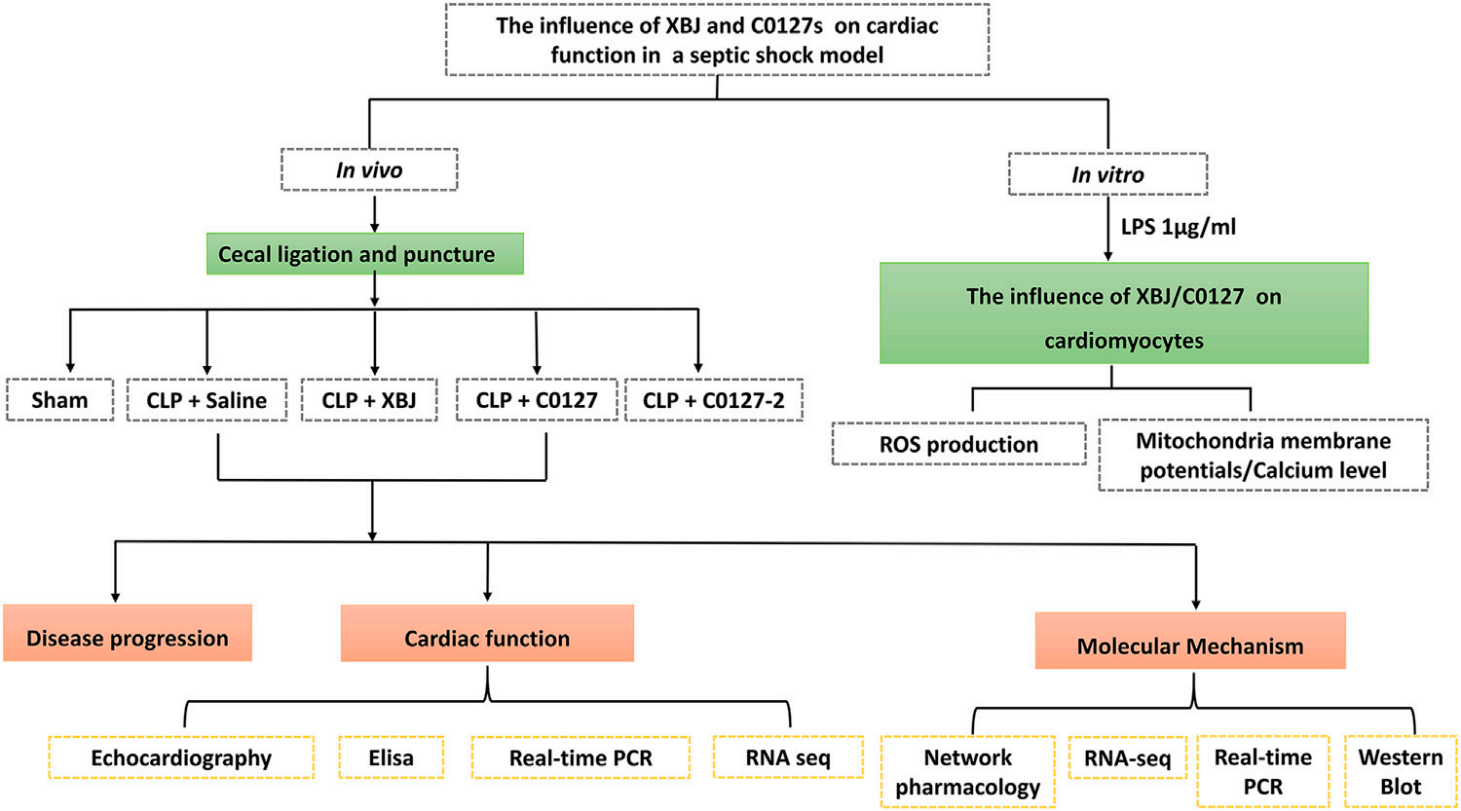
Scheme of the experimental design. *In vivo* and *in vitro* assays were used to determine the impacts of XBJ, C0127, and C0127-2 on infection-induced cardiac dysfunction and potential mechanisms.

**FIGURE 2:**
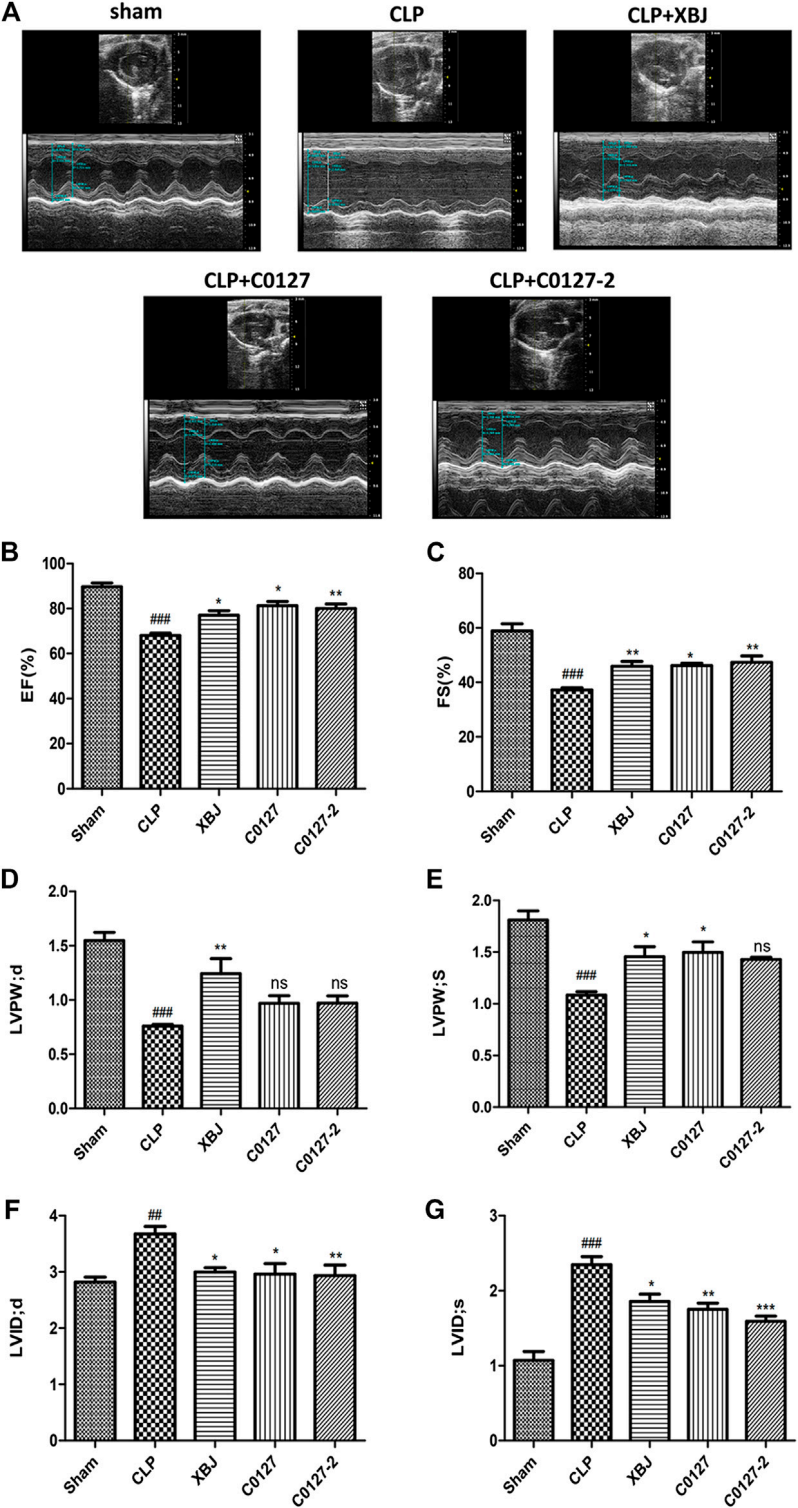
XBJ and key compounds in XBJ protected cardiac function of septic mice. **(A)** Representative images of left ventricular echocardiography. Cardiac performance was determined by echocardiography in different groups as indicated. **(B)** Left ventricularejection fraction (LVEF) %, **(C)** Left ventricular fractional shortening (LVFS) % were measured in M-mode, **(D)** LV posterior wall diastole (LVPWd), **(E)** LV posterior wall systole (LVPWs), **(F)** Left ventricular internal dimensions at diastole (LVIDd), **(G)** Left ventricular internal diameter systole (LVIDs), Results were presented as mean ± SEM (n = 7–10/group). ^#^ *p* < 0.05, ^##^ *p* < 0.01, ^###^ *p* < 0.001 vs. Sham group, *p < 0.05, **p < 0.01 vs. CLP group.

*In vivo* and *in vitro* experiments were designed to determine the influence of XBJ on the cardiac function in septic shock (Figure 1). A septic shock model was established using the cecal ligation and puncture (CLP) procedure. All CLP mice died in 48 h after the procedure and all mice in the sham group survived, indicating the model was established successfully (Supplementary Figure S2B). Cardiac function was measured 24 h after CLP to evaluate the impact of XBJ. CLP significantly compromised cardiac function by reducing ejection fraction (EF%), fractional shortening (FS%), left ventricular posterior wall diastole (LVPWd), and left ventricular posterior wall systole (LVPWs) while increasing left ventricular internal diameter diastole (LVIDd) and left ventricular internal diameter systole (LVIDs), respectively, when compared with the saline-treated sham group. XBJ significantly improved EF, FS, LVPWd, LVPWs, LVIDd, and LVIDs in septic mice (Figure 2).

### XBJ Protected Cardiac Tissue Upon Systemic Infection and Protected Cardiomyocytes From LPS-Induced Mitochondria Ca^2+^ Overload and ROS Production

**FIGURE 3:**
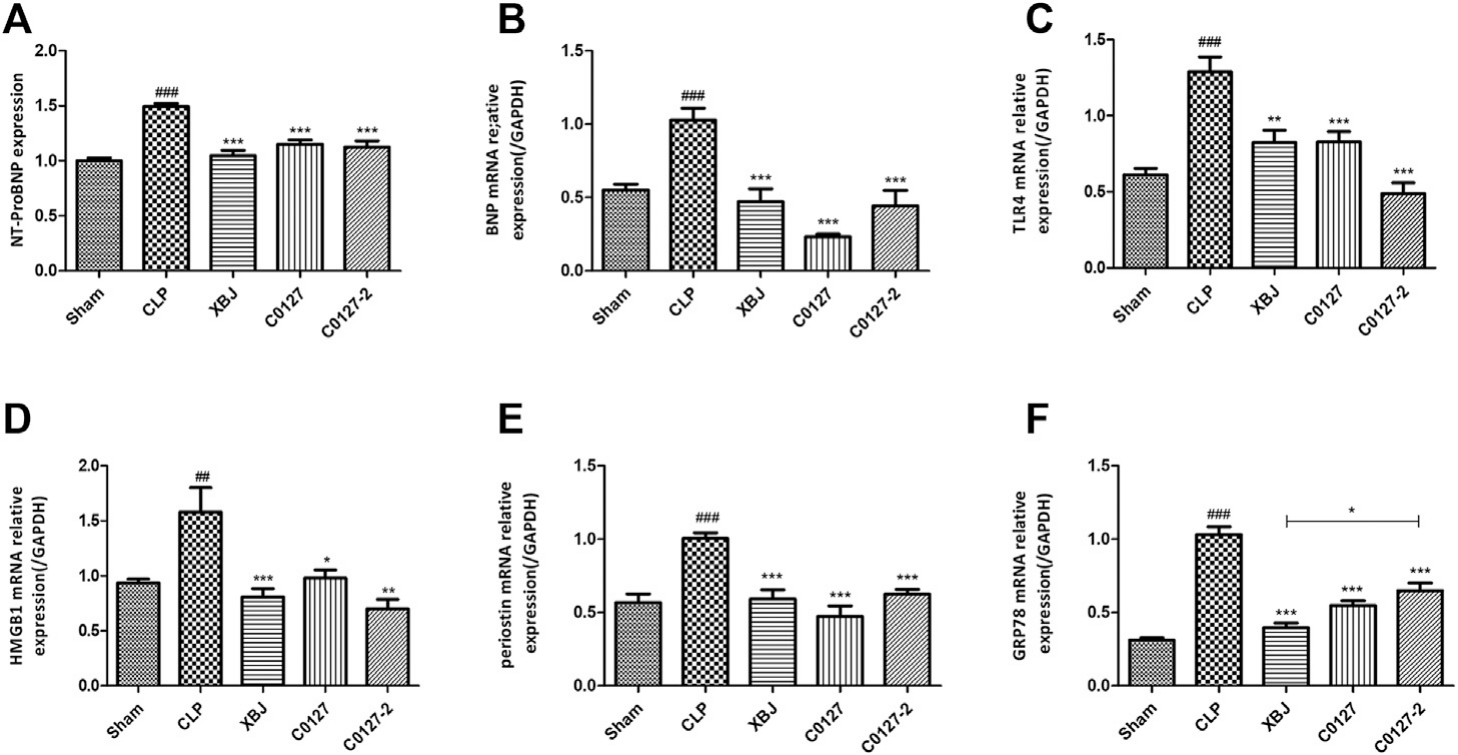
XBJ and C0127s intervention protected hearts in septic mice. **(A)** N-terminal (NT)-pro hormone BNP (NT-ProBNP) levels in different groups were determined by Elisa assay. n = 4/group. mRNA expression of B-type natriuretic peptide (BNP) **(B)**, TLR4 **(C)**, HMGB1**(D)**, Periostin **(E)**, and GRP78 **(F)** in heart tissue of different groups were determined by real-time PCR in heart tissue of different groups. n = 4–5/group. Results were presented as mean ± SEM (n = 4-5/group). ^#^ *p* < 0.05, ^##^ *p* < 0.01, ^###^ *p* < 0.001 vs. Sham group, *p < 0.05, **p < 0.01, ***p < 0.001 vs. CLP group.

**FIGURE 4:**
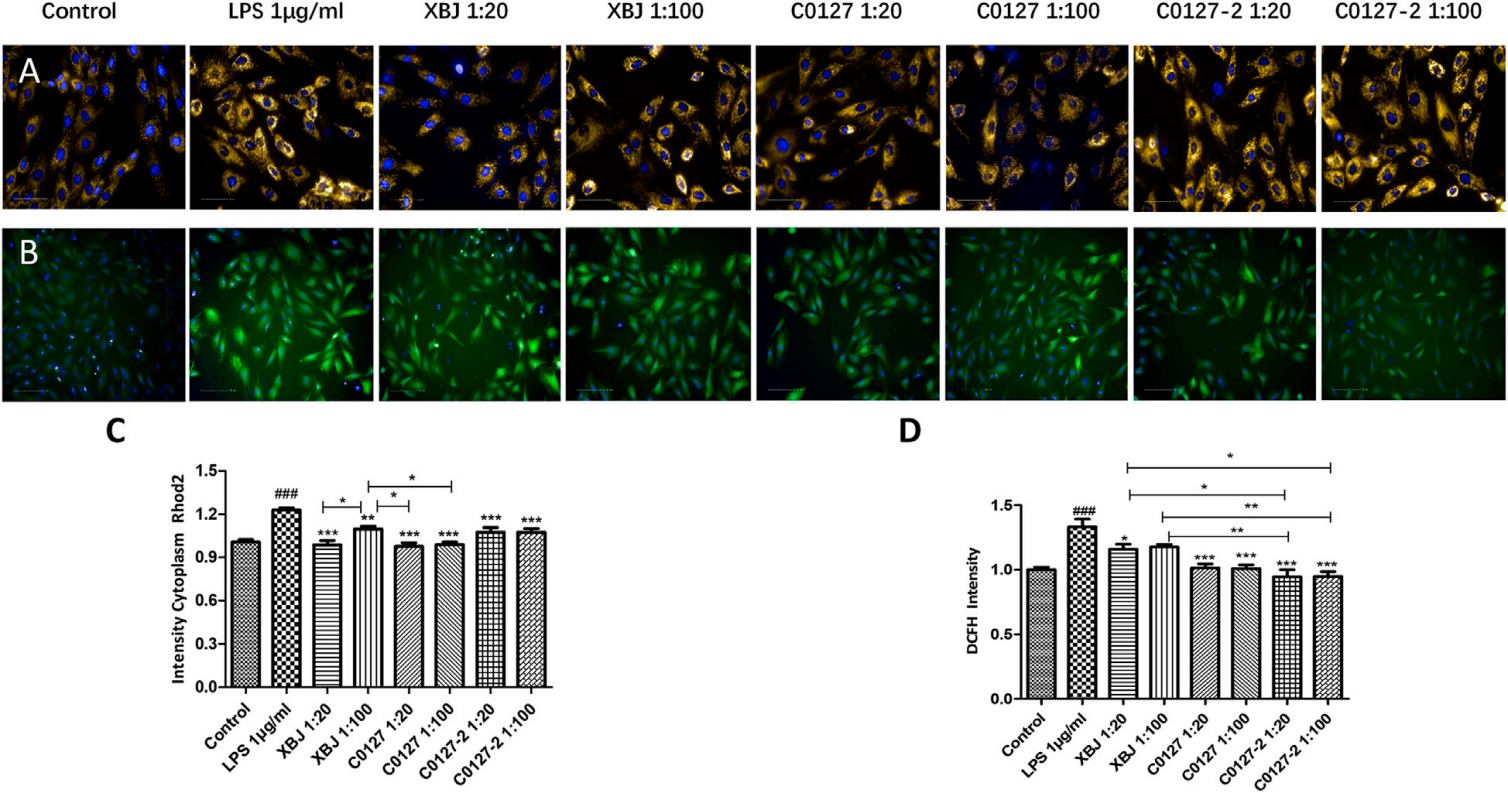
XBJ and C0127s protected mitochondria and reduced ROS production in cardiomyocytes. H9C2 cells were treated with LPS at the presence and absence of XBJ, C0127, and C0127-2 at different concentrations. Rhode2 Florescent probe was used determine Ca^2+^ level in mitochondria and FITC probe was used to detect ROS production in a high-content imaging system. **(A)** Representative images of Ca^2+^ level in mitochondria in different treatments. **(B)** Representative images of ROS level in different treatments. **(C)** Quantification of Ca^2+^ levels in different treatments. **(D)** Quantification of ROS production in different treatments. Results were presented as mean ± SEM. ^#^ *p* < 0.05, ^##^ *p* < 0.01, ^###^ *p* < 0.001 vs. control, *p < 0.05, **p < 0.01, ***p < 0.001 vs. LPS treated cells. Experiments were repeated for 3 times.

H & E staining was conducted to determine the morphology of Cardiac tissue in different groups of mice. Consistent with previous observations by dos Santos et al. (dos Santos et al., 2010), no significant differences in necrosis or fibrosis between CLP and sham mice were detected. However, increased edema associated with some myofibrillary loss was observed in CLP mice (Supplementary Figures S5A,B). XBJ treatments reduced edema and myofibrillary loss in CLP mice (Supplementary Figure S5C). Elisa assay revealed that XBJ significantly reduced N-terminal (NT)-pro hormone BNP (NT-ProBNP) in the peripheral blood (Figure 3A). Consistently, the mRNA expression of B-type natriuretic peptide (BNP), a marker of heart failure (McLean et al., 2008) in the heart tissue was also decreased in the XBJ treated group (Figure 3B). To determine whether XBJ can protect cardiac tissue on the cellular level, *in vitro* assays were conducted on H9C2 cardiomyocytes upon LPS stimulation. XBJ not only reversed LPS-induced Ca^2+^ overload in the mitochondria (Figures 4A,C) but also reduced ROS production (Figures 4B,D).

### XBJ Down-Regulated the Expression of Infection and Inflammation-Related Signaling in the Cardiac Tissue of Septic Mice

**FIGURE 5:**
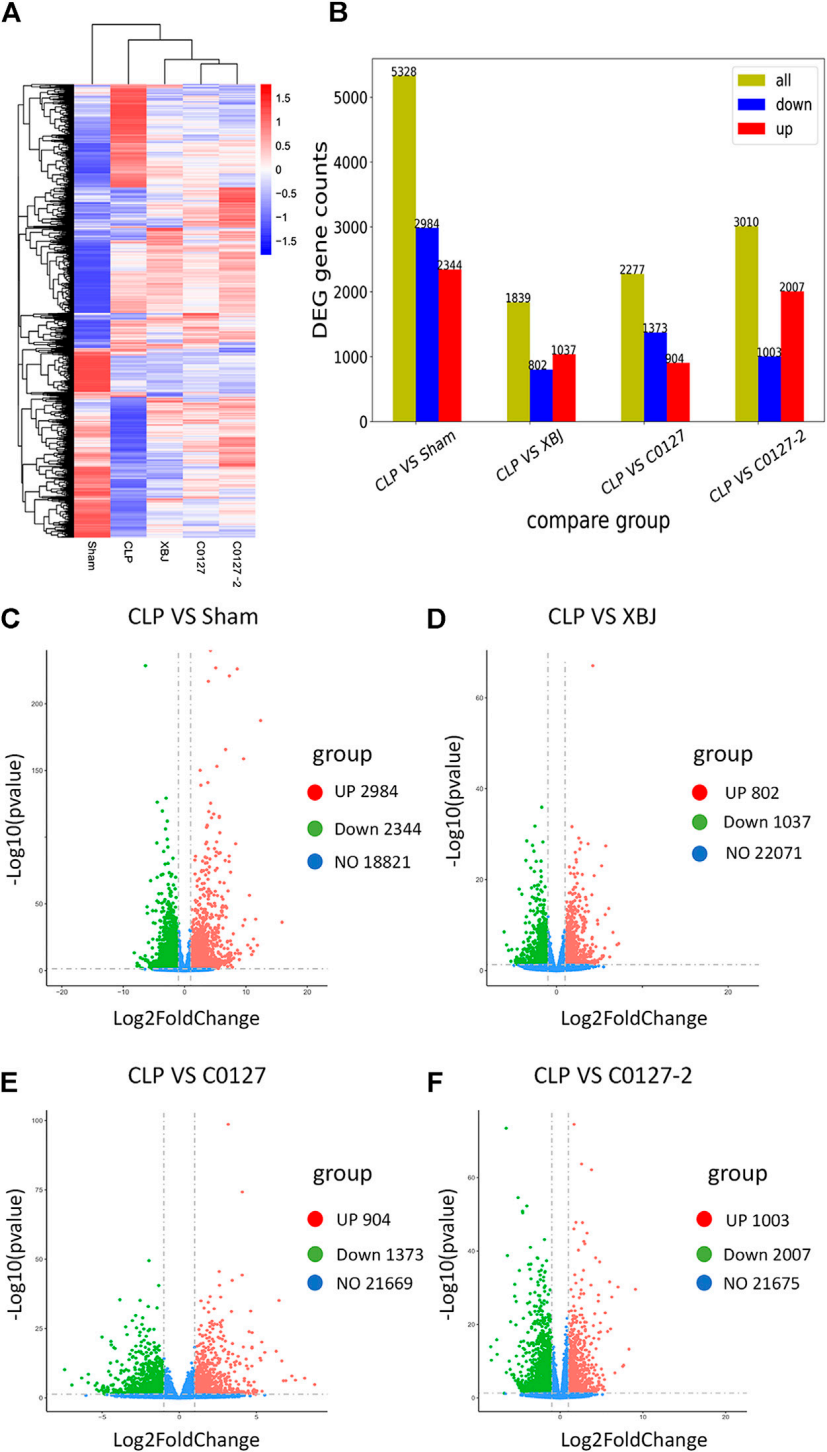
XBJ and C0127s normalized the gene expression profile in the cardiac tissue of septic mice. 24 hs after CLP, mice were euthanized and heart tissue were harvested and subjected to RNA-seq analysis. **(A)** Heat map of the gene expression profiles in different groups. **(B)** Differential gene counts between different groups. **(C–F)** Overall distribution of differentially expressed genes (fold change >2 and *p*-Value <0.05) was reflected by the volcanic map. The X-axis represents the changes of gene expression in different samples and Y-axis represents the statistical significance of the difference in the gene expression. The up-regulated genes were in red and down-regulated genes in green. **(C)** Differentially expressed genes of CLP vs. Sham group; **(D)** Differentially expressed genes of CLP vs. XBJ group; **(E)** Differentially expressed genes of CLP vs. C0127 group; **(F)** Differentially expressed genes of CLP vs. C0127-2 group. n = 4/Group except XBJ and C0127 group (n = 3). *p* value < 0.05, |Log2FoldChange| > 1.

To identify the potential down-stream effectors of XBJ in the cardiac tissue, RNA sequencing was conducted to determine the gene expression profiles of the cardiac tissue in the control and XBJ treated groups. GO and KEGG analysis of the RNA-seq results revealed overwhelming up-regulation of infection and inflammation-related pathways in the CLP group compared to the sham group, including NF-κB, TNF-α, and cytokine-cytokine receptor signaling (data not shown). This is consistent with the results of previous studies (Chen et al., 2018; Villa et al., 1995). XBJ treatment drastically altered the gene expression profile in CLP mice (Figure 5A, Supplementary Figure S3B). It impacted the expression of 1839 genes in CLP mice for over two-fold (Figures 5B,D). GO analysis revealed infection and inflammation-related gene functions were among the top 10 biological processes among the 1839 genes influenced by XBJ, such as response to LPS, response to molecules of bacteria origin, and leukocyte migration (Supplementary Figures S6A,B). KEGG analysis of the XBJ-impacted genes revealed that multiple signaling pathways, including Cytokine-cytokine receptor binding, IL17 signaling, NF-κB, and TNF-α signaling, were related to inflammation and cytokine storm (Supplementary Figures 6C,D), confirming the results of previous studies that XBJ reduced pro-inflammatory cytokine production in the septic mice (Villa et al., 1995; Chen et al., 2018).

### XBJ Down-Regulated the NF-κB Signaling in the Cardiac Tissue of Septic Mice

**FIGURE 6:**
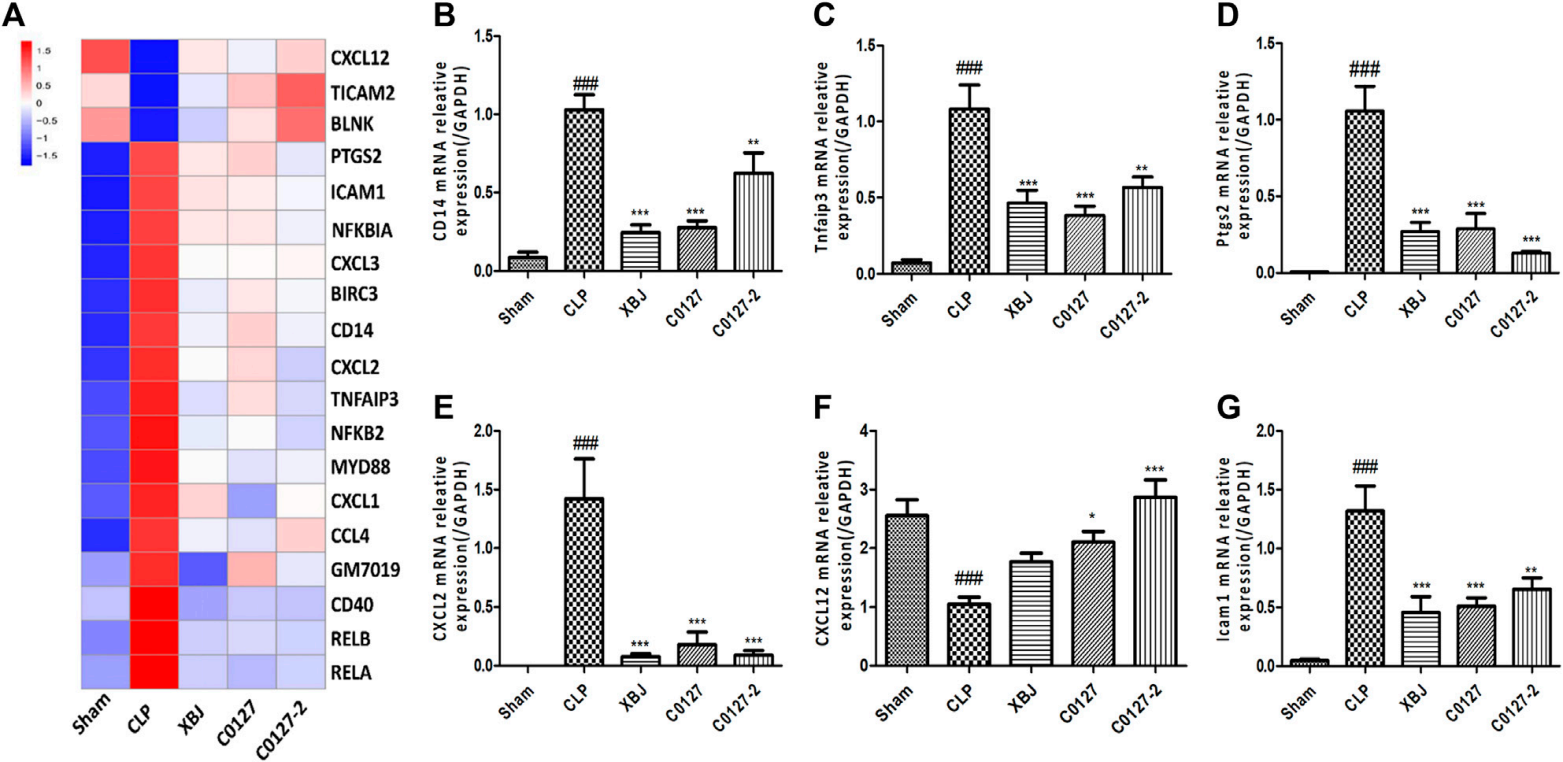
XBJ and C0127s impacted the gene expression of NF-kB signaling pathway in the cardiac tissue of septic mice. **(A)** Heat map of the gene expression influenced by XBJ, C0127, and C0127-2 in NF-κB signaling. **(B–G)** Real-time PCR experiments were conducted to confirm the results of RNA-seq assay. **(B)** CD14; **(C)** TNFAIP3; **(D)** PTGS2; **(E)** CXCL2; **(F)** CXCL12; **(G)** ICAM1. All Real-time PCR experiments were conducted twice. n = 4/group. Results were presented as mean ± SEM (n = 4-5/group). #*p* < 0.05, ##*p* < 0.01, ###*p* < 0.001 vs. Sham group, *p < 0.05, **p < 0.01, ***p < 0.001 vs. CLP group.

As an early signaling activated in sepsis, NF-κB activates the transcription of a series of proinflammatory cytokines to activate cytokine storm in septic shock, including TNF-α, IL-1β, and IL-12 (Hotchkiss et al., 2016). Several groups reported that XBJ regulates NF-κB signaling which activates a series of pro-inflammatory cytokines, including TNF-α, IL-6, and IL-1 (Jiang et al., 2013; Chen et al., 2018). Our RNA-seq assay revealed that XBJ regulated NF-κB signaling spanning from the receptor, intracellular effectors, transcription factor, and downstream targets (Figure 6A). The expression of at least six genes in NF-κB signaling was confirmed by Real-time PCR assay, including CD14, CXCL2, and Ptgs2 (Figures 6B–G).

### XBJ Down-Regulated TNF-α Signaling and the Expression of Proinflammatory Cytokines and Cytokine Receptors in the Cardiac Tissue of Septic Mice

**FIGURE 7:**
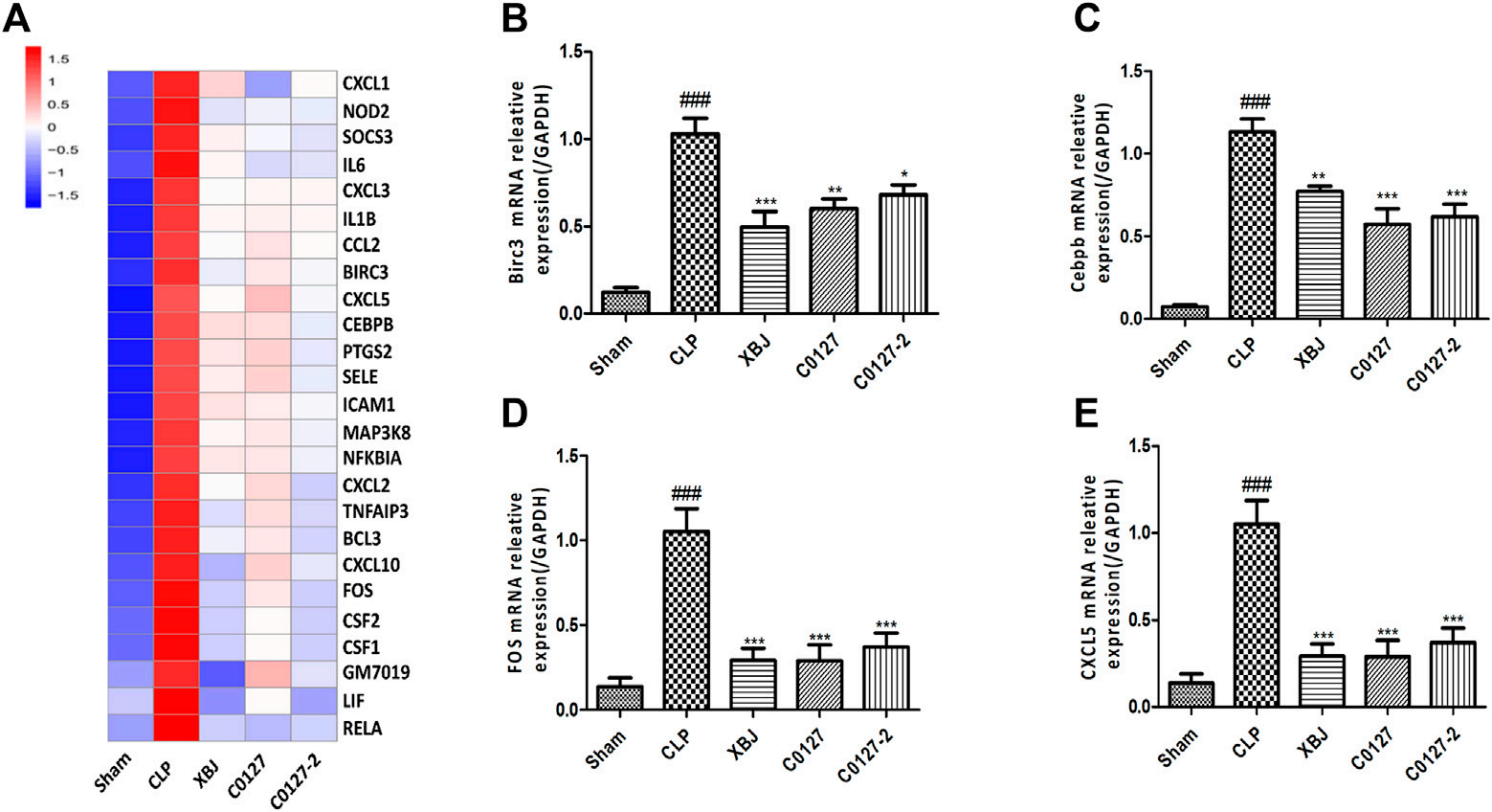
XBJ and C0127s down-regulated TNF-α signaling in the cardiac tissue of septic mice on mRNA level. **(A)** Heat map of the gene expression influenced by XBJ and C0127s in TNF-α signaling in the RNA-seq assay. **(B–E)** Real-time PCR experiments were conducted to confirm the results of RNA-seq assay. B. Birc3; C. C/EBPβ; D. FOS; E. CXCL5. n = 4/group. All Real-time PCR experiments were conducted twice. Results were presented as mean ± SEM (n = 4/group). #*p* < 0.05, ##*p* < 0.01, ###*p* < 0.001 vs. Sham group, *p < 0.05, **p < 0.01, ***p < 0.001 vs. CLP group.

**FIGURE 8:**
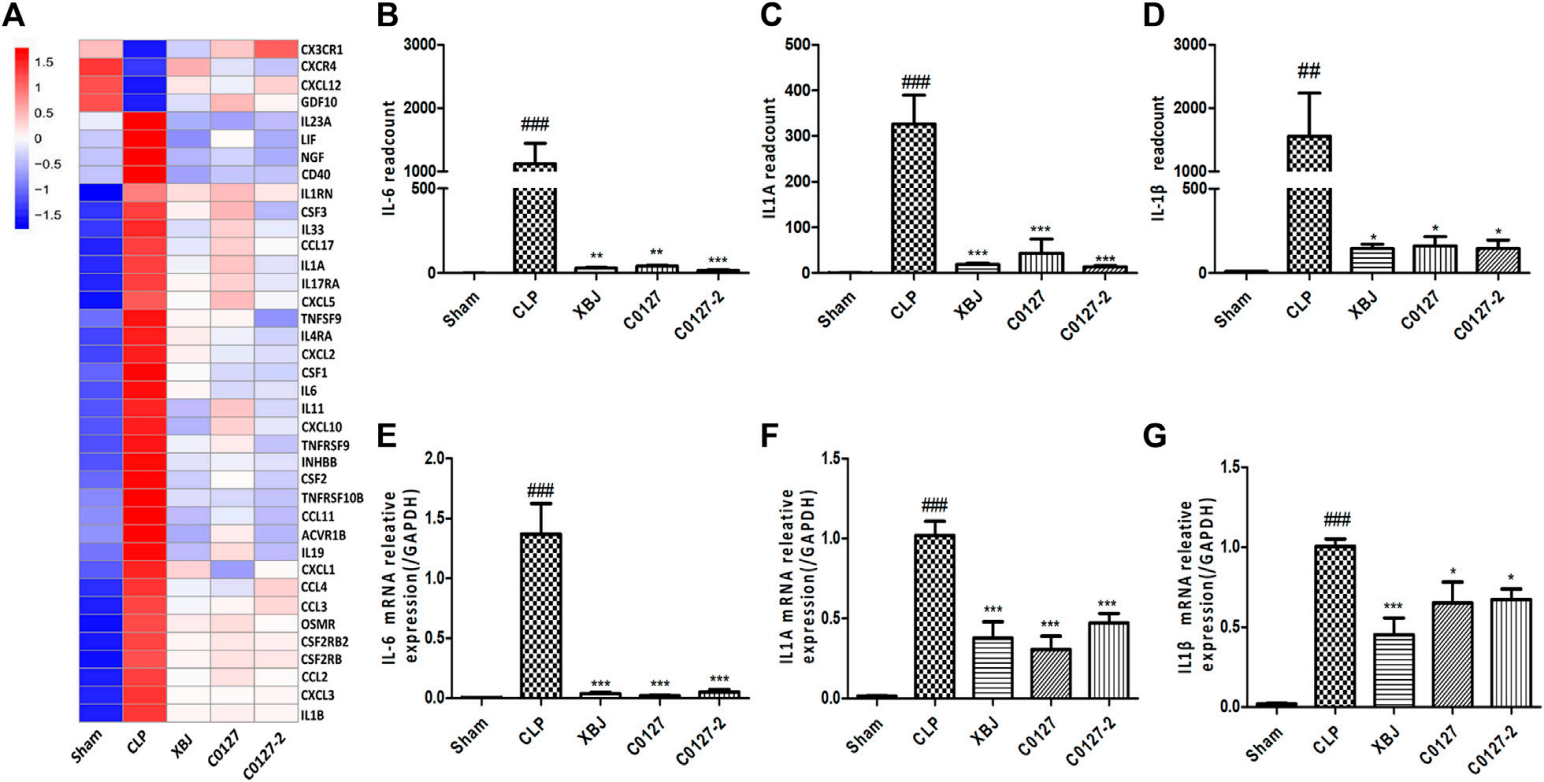
XBJ and C0127s down-regulated the mRNA expression of pro-inflammatory cytokines and cytokine receptors in the cardiac tissue after systemic infection. **(A)** Heat map of the gene expression influenced by XBJ, C0127, and C0127-2 in Cytokine-cytokine receptor interaction in the RNA-seq assay. **(B–D)** The expression of pro-inflammatory cytokines, IL-6, IL-1α, and IL-1β were detected in the cardiac tissue of different groups of mice. B. IL-6; C. IL-1α; D. IL-1β. **(E–G)** The expression of pro-inflammatory cytokines in the cardiac tissue of different groups of mice was determined by Real-time PCR. E. IL-6; F. IL-1α; G. IL-1β. n = 4-5/group. Results were presented as mean ± SEM (n = 4-5/group). #*p* < 0.05, ##*p* < 0.01, ###*p* < 0.001 vs. Sham group, *p < 0.05, **p < 0.01, ***p < 0.001 vs. CLP group.

**FIGURE 9:**
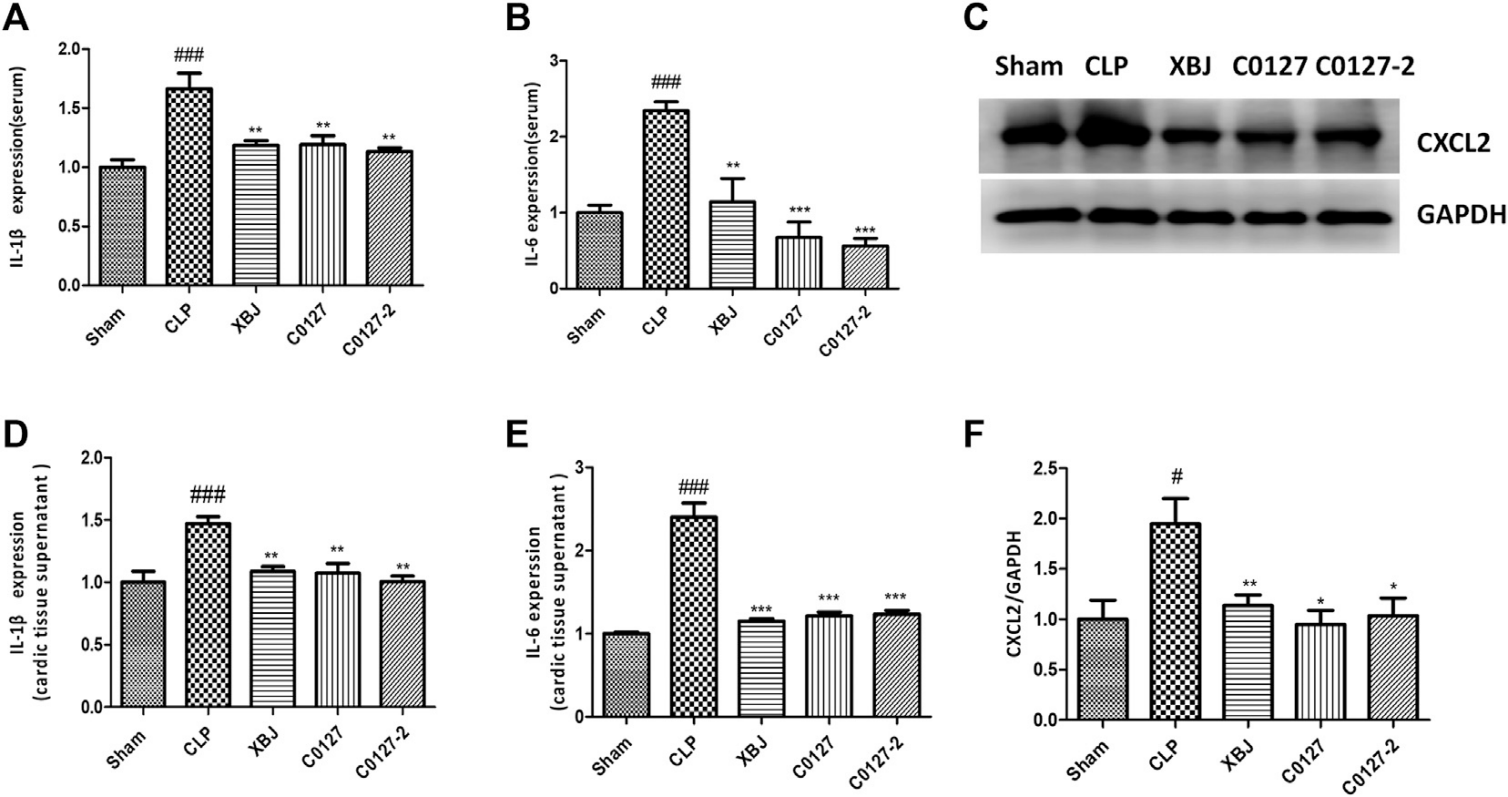
XBJ and C0127s down-regulated the protein expression of pro-inflammatory cytokines/chemokines in the septic mice. **(A,B)** The serum levels of **(A)** IL-1β and **(B)** IL-6 were measured by ELISA. Each bar was represented as mean ± SEM (n = 5); **(D,E)** The levels of **(D)** IL-1β and **(E)** IL-6 in heart tissues were measured by ELISA. Each bar was represented as mean ± SEM (n = 5); **(C,F)** The expression of CXCL2 in the heart tissue of different groups was determined by western blot. Each bar was represented as mean ± SEM (n = 5/group). #*p* < 0.05, ##*p* < 0.01, ###*p* < 0.001 vs. Sham group, *p < 0.05, **p < 0.01, ***p < 0.001 vs. CLP group. Experiments were repeated for three times.

Expressed early in the pathogenesis of sepsis, TNF-α is a potential therapeutic target in sepsis (Hotchkiss et al., 2016; Huang et al., 2019). XBJ reduced TNF-α production in patients and pre-clinical models (Chen et al., 2018; He et al., 2018). RNA-seq analysis did not reveal a significant difference of TNF-α expression between CLP and XBJ treated group in the cardiac tissue on RNA level 24 h after CLP (data not shown). However, the expression of multiple TNF-α downstream targets was significantly reduced in XBJ treated septic mice, compared with the CLP group (Figure 7A). The expression of more than six genes in TNF-α signaling was confirmed with real-time PCR, including intracellular protein Birc3 (cIAP2), transcription factor C/EPBβ, TNF-α downstream targets ICAM1, CXCL2, CXCL5, FOS, and PTGS2 (COX2) (Figures 7B–G). Some of them were molecules in NF-κB signaling (ICAM1, PTGS2 (COX2) (Aronoff, 2012), and Birc3) (Figure 6). Besides, XBJ impacted the expression of more than 50 Cytokine-cytokine receptor interaction molecules in the cardiac tissue of septic mice, including key pro-inflammatory cytokines causing tissue damages, such as IL-6, IL-1α and IL-1β (Figures 8A–D). The down-regulation of IL-6, IL-1α, and IL-1β by XBJ was further confirmed by Real-time PCR (Figures 8E–G). Elisa assay confirmed that XBJ inhibited IL-6 and IL-1β in the serum and cardiac tissue (Figures 9A,B,D,E). Western Blot analysis validated that XBJ inhibited the expression of CXCL2 in the cardiac tissue (Figures 9C,F).

### C0127s Represented XBJ Function in Improving the Survival and Protecting the Cardiac Function in a Septic Shock Model

So far, the potential material base of XBJ in sepsis management and infection-related cardiac protection was not clear. Since a combination of four compounds in XBJ (C0127) prevented Candida-induced sepsis and kidney failure (Shang et al., 2019), we hypothesized that XBJ attenuates infection-induced cardiac dysfunction and C0127 plays a major role in managing septic shock and infection-induced cardiac depression. Like XBJ, C0127 and C0127-2 rescued about 35% of septic mice from acute death. There was no significant difference among the treatment groups (Supplementary Figure S2B).

### C0127s Protected the Cardiac Tissue During Systemic Infection Partially by Preventing LPS Induced Injuries in Cardiomyocytes

Echocardiography was used to address the question of whether active ingredients in XBJ render cardiac protection in septic mice. C0127s showed similar effects as XBJ in most parameters, including LVIDd, LVIDs, LVPWs, LVEF %, and LVFS (Figure 2). Nonetheless, C0127s did not significantly alter the LVPWd (Figure 2D). C0127-2 treatment did not significantly impact the LVPWs (Figure 2E).

Consistently, C0127s reduced N-terminal (NT)-pro hormone BNP (NT-ProBNP) in the peripheral blood (Figure 3A). They also reduced the mRNA expression of B-type natriuretic peptide (BNP) (McLean et al., 2008) in the cardiac tissue of septic mice (Figure 3B). In addition, the expression of TLR4, its downstream effector HMGB1, periostin, and GRP78 (an effector of ER stress (Shang et al., 2019)) were moderately decreased in C0127s treated groups (Figures 3C–F). H&E staining showed that C0127s reduced edema and myofibrillary loss in CLP mice. There were no obvious differences between XBJ and C0127s treated groups (Supplementary Figures S5C–E).

On the cellular level, C0127s reversed Ca^2+^ overload in the mitochondria (Figures 4A,C) and reduced ROS production (Figures 4B,D) in LPS-stimulated H9C2 cells, suggesting the active compounds in XBJ protect cellular functions of cardiomyocytes.

### Active Ingredients in XBJ Down-Regulated Gene Expression in Infection and Inflammation-Related Signaling in the Cardiac Tissue of Septic Mice

We conducted an RNA sequencing to determine the impacts of C0127s on the expression profile of the cardiac tissue in septic mice. The gene expression patterns were consistent in each group (Supplementary Figure S3B). Similar to XBJ intervention, C0127s shifted the gene expression profile of the CLP group (Figure 5A, Supplementary Figure S3B). C0127s treatments triggered more changes in the gene expression profile of CLP mice, comparing to the XBJ treated group (Figure 5B). Notably, 952 genes were differentially regulated by XBJ and C0127s for >2 fold, revealing similar expression trends as the sham group (Supplementary Figure S3A). We further analyzed these genes using GO and KEGG databases as references.

**FIGURE 10:**
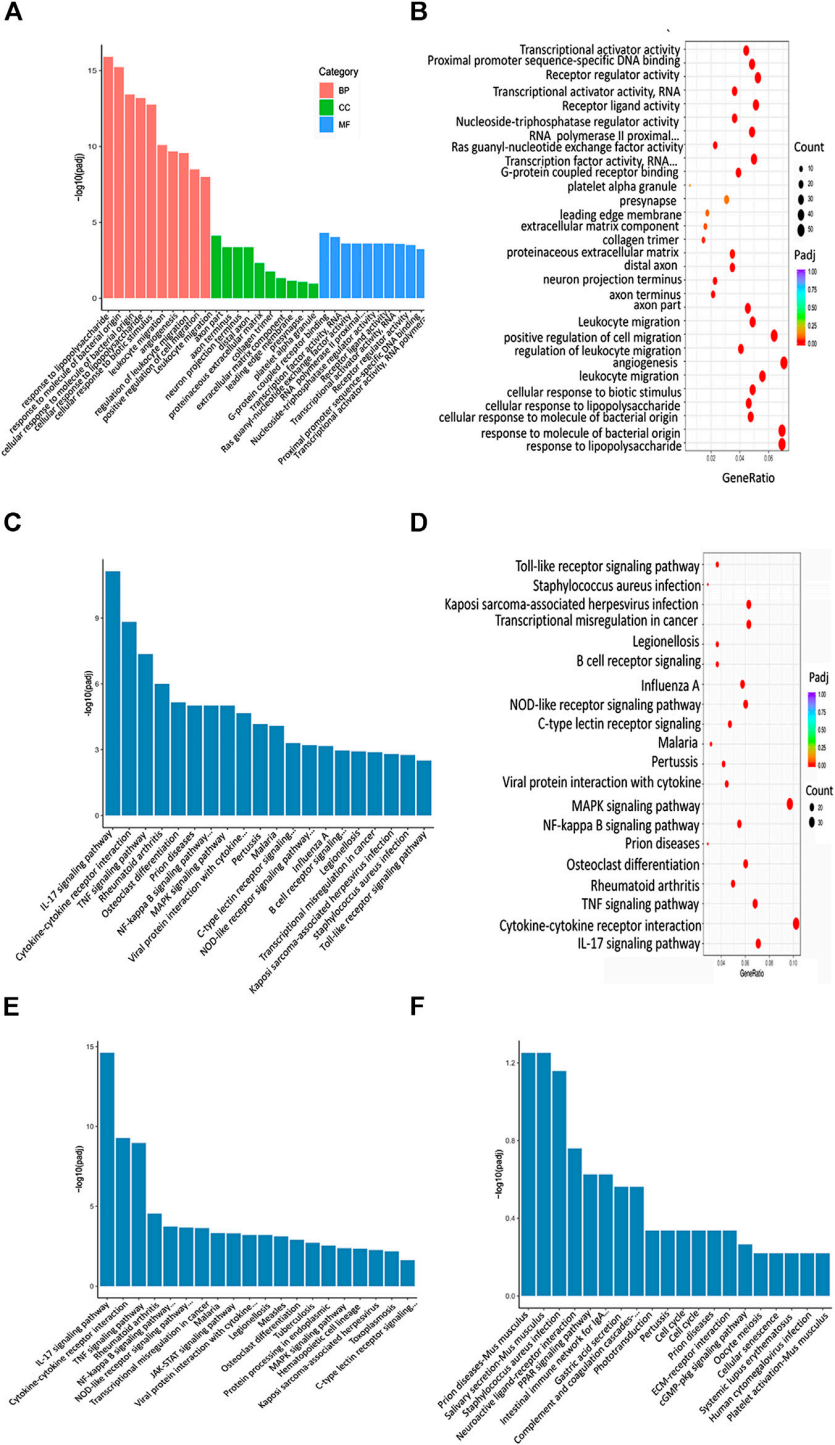
XBJ and C0127s regulated infection and inflammation related signaling in septic shock. **(A,B)** GO analysis of the top 30 functions impacted by XBJ and C0127s. BP, biological process, CC, cellular component, MF, molecular function. Padj: P value adjusted. **(C,D)** The KEGG 676 analysis of top 20 pathways of the 952 genes impacted by XBJ and C0127s for >2 fold. The pathways/functions were ranked from left to right by −log (P value adjusted). **(E,F)** KEGG analysis of the top signaling pathways up-regulated **(E)** or down-regulated **(F)** by XBJ and C0127s were presented.

GO analysis revealed infection and inflammation-related gene functions were among the top 10 biological processes in the 952 genes, such as response to LPS, response to molecules of bacteria origin, and leukocyte migration (Figures 10A,B). Interestingly, the top three signaling pathways impacted by C0127s among the 952 genes, such as IL17 signaling, Cytokine-cytokine receptor binding, and TNF-Α signaling pathway, were also the top pathways impacted by XBJ (Figures 10C,D). Like XBJ, C0127s significantly down-regulated gene expression in the infection and proinflammatory signaling (Figure 10E). PPAR signaling was up-regulated by XBJ and C0127s (Figure 10F).

### Active Ingredients in XBJ Down-Regulated the Expression of NF-kB Target Genes in the Cardiac Tissue of Septic Mice

Like XBJ, C0127s also regulated NF-κB signaling spanning from the receptor, intracellular effectors, transcription factors, to the downstream targets (Figures 6A,B, and Supplementary Tables). Twenty genes in NF-κB signaling which were impacted by XBJ, were also down-regulated by C0127s in the cardiac tissue of the septic mice (Figure 6A). The influences of C0127s on the expression of at least six genes in the NF-κB signaling were confirmed by the Real-time PCR assay, including CD14, CXCL2, and Ptgs2 (Figures 6B–G).

### Active Ingredients in XBJ Down-Regulated TNF-α Signaling and the Expression of Pro-inflammatory Cytokine and Cytokine Receptors in the Cardiac Tissue of Septic Mice

Interestingly, C0127s not only mimicked the effect of XBJ on sepsis progression and cardiac protection, they also regulated the expression of the same genes in TNF-α signaling in the cardiac tissue (Figure 7A). The expression of 24 genes in TNF-α signaling and 38 Cytokine-cytokine receptor interaction molecules which were influenced by XBJ, were also similarly impacted by C0127s in the cardiac tissue in septic mice (Figures 7A, 8A, and Supplementary Tables). The impacts of C0127s on at least six genes in TNF-α signaling were confirmed with real-time PCR, including intracellular protein Birc3 (cIAP2), transcription factor C/EPBβ, TNF-α downstream targets ICAM1, CXCL2, CXCL5, FOS, and PTGS2 (COX2) (Figures 7B–E, 6). Some of them were also regulated by XBJ and C0127s in NF-κB signaling (ICAM1, PTGS2 (COX2) (Aronoff 2012), and Birc3). Similar to XBJ, C0127s significantly down-regulated the expression of IL-6, IL-1α, and IL-1β in the cardiac tissue of septic mice (Figures 8B–D). These results were also confirmed with real-time PCR (Figures 8E–G).

### C0127s Inhibited the Septic Shock-Induced Myocardial Production of Inflammatory Cytokines/Chemokines

To further evaluate the impacts of C0127s on the inflammatory cytokine/chemokine production, we determine the inflammatory cytokines/chemokines in the serum and cardiac tissue of CLP mice using ELISA and Western blot. CLP induced a drastic increase of IL-6 and IL-1β in the peripheral blood and the cardiac tissue 24 h after the CLP procedure. C0127s pretreatment significantly inhibited the IL-1β and IL-6 production (Figures 9A,B,D,E). Western blot further confirmed that C0127s inhibited CXCL2 production in the cardiac tissue (Figures 9C,F). Overall, attenuating cytokine storm by XBJ and its key ingredients may play a major role in protecting cardiac function in septic shock.

## Discussion

In this study, we focused on the influence of XBJ on infection-induced cardiac dysfunction and the potential molecular mechanism of its effects. We found XBJ and C0127s improved survival and cardiac function after systemic infection. RNA-seq analysis revealed that C0127s exerted similar functions as XBJ in controlling the cytokine storm and inflammation-related signaling pathways, including NF-κB and TNF-α signaling. Real-time PCR experiments confirmed the results of RNA sequencing on the mRNA level. XBJ and C0127s protected the cardiac tissue after the septic shock by normalizing the expression of pro-inflammatory cytokines (IL-6 and IL-1β), chemokines (CXCL2, CXCL5, and CXCL12), and molecules in TNF-α and NF-κB signaling. XBJ and C0127s also protected cardiac function on the cellular level by reducing LPS-induced ROS production and calcium overload in mitochondria of cardiomyocytes. These findings partially explained the protective effects of XBJ in septic shock and severe COVID-19 patients.

### C0127s Improved the Survival of Septic Mice

XBJ contains compounds derived from five different Chinese medicinal herbs (Cheng et al., 2016; Chen et al., 2018; Shang et al., 2019). It is not clear which compounds in XBJ played major roles in managing sepsis and septic shock. Our previous work showed C0127, a 4-compound formula, mimicked the effect of XBJ in preventing systemic Candida infection (Shang et al., 2019), indicating high-concentration compounds in XBJ may execute its major functions *in vivo*. Paeoniflorin (Pae) and Hydroxysafflor yellow A (HSYA) are the two compounds with the highest and 2nd highest concentrations in XBJ (Li et al., 2020a) (The chemical structures of compounds in XBJ (including Pae and HSYA) were shown in Supplementary Figure S1). They are key indicators for the quality control of this injection (Zhang et al., 2018; Shang et al., 2019). How these two compounds affect septic shock and infection-induced cardiac injuries/dysfunction was not well-understood. XBJ and C0127s significantly improved survival in a murine septic shock model. However, there was no significant difference between the intervention groups, indicating Pae and HSYA are major active compounds in XBJ to manage septic shock.

### C0127s Improved the Cardiac Function of Septic Mice

Limited studies were conducted on cardiac dysfunction in systemic infection. Dos Santos et al. observed the down-regulation of PPAR signaling in the cardiac tissue of a murine CLP model (dos Santos et al., 2010). Consistently, our studies revealed XBJ and C0127s significantly up-regulated PPAR signaling which is critical for normal cardiac function (Supplementary Figures 4A,B).

From septic shock to COVID-19, systemic infections compromise cardiac function (dos Santos et al., 2010; Zheng et al., 2020). Patients with cardiovascular conditions are more vulnerable to the assaults of systemic bacterial infection (Zhu et al., 2020). Protecting the cardiovascular function of these high-risk patients may save tens of thousands of lives. Our IPA network pharmacology analysis predicted that XBJ, a formula of Chinese medicine activating circulation and removing stasis, may improve cardiovascular function. Some publications also provided evidence for the role of XBJ in regulating cardiovascular function (Zhai and Guo, 2016a).

In a well-established septic shock-induced myocardial dysfunction model, we observed similar phenotypes as previously described (dos Santos et al., 2010). Overall, C0127s showed similar effects as XBJ in restoring cardiac functions in the septic mice (Figure 2). No significant differences were observed between C0127 and C0127-2 group, suggesting paeoniflorin and HSYA are the core compounds in XBJ for cardiac protection.

Two previous studies suggested paeoniflorin rendered cardiac protection in LPS-induced endotoxemia models (Zhai and Guo, 2016b; Liu et al., 2016). Liu et al. found 12 mg/kg paeoniflorin once/day reduced plasma CK-MB (a biomarker of severe heart damage) to normal level 72 h after the LPS administration (5 mg/kg) in a rat model (Liu et al., 2016). Zhai and Guo revealed that paeoniflorin reduced pro-inflammatory cytokine production in the cardiac tissue of a LPS-induced cardiac-dysfunction mouse model (Zhai and Guo, 2016b). Treating mice with paeoniflorin 15 mg/kg/day for 3 days before LPS injection (10 mg/kg) improved cardiac function in endotoxemic mice and reduced LPS-induced Leukocyte infiltration in the cardiac tissue. It reduced LPS-induced cytokine production on mRNA level (in the cardiac tissue) and protein level (in the serum), including IL-6, TNF-α, IL-12, IL-1β, and IFNγ (Zhai and Guo, 2016b). These results indicated that paeoniflorin may play an important role in XBJ and C0127s to reduce the cytokine expression in the cardiac tissue of the septic mice in our study.

HSYA, which promotes angiogenesis, was known for its anti-thrombosis effect. It is used to treat stroke and ischaemic cardiovascular diseases in China. HSYA (240 mg/kg/day) rescued CD4 T cell apoptosis in a mouse CLP model. HSYA (240 mg/kg/day) also protected mice from LPS-induced lung injury (Liu et al., 2014b; Wang et al., 2017). Another study discovered that HSYA (25 mg/kg/day) stimulated angiogenesis to improve cardiac function in a murine ischaemic model (Zou et al., 2018). This effect was mediated by nucleolin, a DNA-binding molecule that regulates angiogenesis. Huber et al. found HSYA can enter cardiac myocytes to reduce anoxia/reoxygenation-induced damage by interacting with the mitochondrial permeability transition pore *in vitro* (Huber et al., 2018).

So far, it is not clear whether HSYA can improve survival and cardiac function in CLP mice. Overall, our and other groups’ results suggested paeoniflorin and HSYA may synergistically improve cardiac function during systemic infection by reducing cytokine/chemokine production.

Biochemical and molecular analysis *in vivo* and *in vitro* further confirmed the results of echocardiography. C0127s not only reduced NT-pro-BNP on the protein level but also inhibited other drivers of cardiac dysfunction, such as IL-6, IL-1β and CXCL2 expression on mRNA and protein level (Figures 3A,B, 8, 9) (McLean et al., 2008; Zhu et al., 2020). They also reduced LPS induced ROS production and Calcium overload in the mitochondria of cardiomyocytes (Figure 4).

The increased GRP78 expression in the cardiac tissue of CLP mice was a response to the CLP- induced acute cardiac stress (Figure 3F). This result echoed findings by a recent study (Bi et al., 2018). Bi et al. revealed that overexpressing GRP78 protected myocytes from I/R-induced cell death by activating AKT signaling. It is likely that the decreased expression of GRP78 after XBJ and C0127s treatment indicated the reduced ER stress an improved cardiac function in CLP mice.

### XBJ and C0127s Directly or Indirectly Attenuate Cytokine Storm to Protect Cardiac Function and Improve Survival in Septic Shock

To explore the molecular mechanism of XBJ on infection-induced cardiac dysfunction, we conducted RNA–seq assay in different groups of mice to determine the impact of XBJ and C0127s on the gene expression profile of septic hearts. GO and KEGG analysis revealed that XBJ and C0127s regulate infection and inflammation related signalings in septic mice (Figure 10). The most pronounced change in the gene expression profile of XBJ-treated CLP mice was the pan-reduction of gene expression related to cytokine storms during infection (Figure 10 and Supplementary Figures S6C,D). Not only pro-inflammatory cytokines such as IL-1α, IL-1β, and IL-6 but also the cytokine receptors, cellular mediators, downstream transcription factors, and target genes were down-regulated by the treatments (Figures 6, 7, 8). Consistently, C0127s also down-regulated the same groups of pro-inflammatory cytokines and receptors (Figures 6, 7, 8), indicating paeoniflorin and HSYA are the core compounds in XBJ to regulate the local expression of inflammation-related signaling in the cardiac tissue of septic mice. CD40-NF-κB signaling pathways is a representative signaling pathway significantly down-regulated by XBJ and C0127s. CD154, a marker of cardiac ischemia and the ligand of CD40, was often elevated in septic patients (McLean et al., 2008). Both CD154 and CD40 are potential therapeutic targets of cardiovascular disease (Bosmans et al., 2020). In our study, XBJ and C0127s significantly reduced the expression of CD40. In addition, the expression of NF-κB p65 subunit (RELA) was also reduced upon XBJ and C0127s treatments (Figure 6A). This result was consistent with a previous report that XBJ reduced NF-κB protein expression in the lung (He et al., 2018).

Additionally, XBJ and C0127s down-regulated the mRNA and protein expression of NF-κB targets, IL-6 and IL-1β, in the cardiac tissue. Given that IL-6 is a marker of cardiac ischemia (McLean et al., 2008), XBJ and C0127s may improve circulation in the cardiac function of septic mice. These results suggested Pae and HSYA are core compounds in XBJ to regulate pro-inflammation cytokine production in the cardiac tissue.

### The Influence of XBJ and C0127s on CXCL2

CLP stimulated the expression of a series of chemokines in the cardiac tissue, including CXCL1, CXCL2, and CXCL5. XBJ and C0127s significantly down-regulated the expression of these chemokines (Figures 7A, 8A, 9A). Real-time PCR confirmed these effects on CXCL2, CXCL5, and CXCL12 in the cardiac tissue (Figures 8E, 9C,F). Western blot analysis further validated that XBJ and C0127s inhibited CXCL2 protein expression (Figures 4E,F). Consistent with our results, Li et al., found XBJ down-regulated CXCL2 expression in a methicillin-resistant *Staphylococcus aureus* (MRSA) infection model (Li et al., 2020a).

CXCL2, which is also known as Macrophage Inflammatory Protein 2 (MIP-2) and GRO2, is a small cytokine that is expressed in a spectrum of tissues and organs including the heart (https://www.ncbi.nlm.nih.gov/gene/20310). CXCL1 (GRO1) shares 90% of sequences with CXCL2. Both CXCL1 and CXCL2 can be secreted by monocytes and neutrophils at the site of inflammation. They function through the chemokine receptor CXCR2 to act as chemotactic agents for leukocytes and hematopoietic cells (Sawant et al., 2020).

Two studies revealed that CXCL2 polymorphism is related to the severity of sepsis in patients, suggesting CXCL2 playing an important role in the development of severe sepsis (Flores et al., 2006; Villar et al., 2007). IL-17, NF-κB, and IL-1β can stimulate CXCL2 expression in pathological conditions (Guo et al., 2020). CXCL2 is believed to be a critical therapeutic target in myocardial infarctions (MI) (Guo et al., 2020). A high level of CXCL2 worsens MI by stimulating neutrophil-mediated cardiac injuries. Interventions that down-regulate CXCL2 relieved neutrophil-mediated cardiac injuries (Montecucco et al., 2010; Montecucco et al., 2013; Mylonas et al., 2017). Our results suggested XBJ improves cardiac function partially by reducing CXCL1 and CXCL2 production to limit the neutrophil-mediated cardiac injury. Interestingly, C0127s also down-regulated the mRNA and protein expression of CXCL2 (Figures 6A,E, 9C,F). How XBJ and C0127s regulate CXCL2 signaling in different cell types remains to be determined.

The balance of CXCL12 (SDF-1) and CXCL2 chemokines favors neutrophil retention in the BM in steady-state conditions (Eash et al., 2010; Day and Link, 2012). Under stress situations such as infections, G-CSF induces massive neutrophil egress into the circulation. G-CSF inhibits SDF-1 expression in the BM. It also causes cleavage of surface CXCR4 (a CXCL12 receptor) on neutrophils, disrupting SDF-1/CXCR4 signaling, which leads to their mobilization (Day and Link, 2012). Guan et al. found that combining CTCE (a peptide analogue of CXCL12) and antibiotics improves survival and neutrophil recruitment in a murine sepsis model (Guan et al., 2014). However, Liehn et al.’s results raised a concern about the expression of endogenous CXCL12 on cardiovascular function in a myocardial infarction (MI) model (Liehn et al., 2011). Increased expression of CXCL12 in the cardiac tissue after XBJ and C0127s treatments may indicate a balanced neutrophil mobilization or improved clearance of infection (Figure 6F). More studies need to be conducted to explain this phenomenon in the cardiac tissue.

### The Implication of This Study for COVID-19 Management

COVID-19 patients having cardiac complications are suffering the second-highest mortality rate in the global pandemic. Cardiovascular complications occurred in up to 16% of critically ill COVID-19 patients (Li et al., 2020a; Zhu et al., 2020). Myocardial injury is a leading cause of severe illness and mortality in SARS-CoV-2 infection (Tersalvi et al., 2020; Zheng et al., 2020). About 5% of COVID-19 patients undergo septic shock and multiple organ failure (Russell et al., 2020; Zhu et al., 2020). Stunningly, 70% of patients who died of COVID-19 had septic shock (Hantoushzadeh and Norooznezhad, 2020). Sharing multiple traits with septic shock, severe COVID-19 was defined as viral sepsis (Shankar-Hari et al., 2016; Li et al., 2020a).

High levels of IL-6 and IL-1β drive the cytokine storm in severe COVID-19 patients, causing organ injuries and MODS. Deceased COVID-19 patients had significantly higher IL-6 expression than the survivors of COVID-19 (Ruan et al., 2020; Zhou et al., 2020). As a promising therapeutic target of severe COVID-19, IL-6 was downregulated by XBJ in clinical studies and pre-clinical studies.

Approved to treat COVID-19 in China, XBJ was effective in managing moderate and severe COVID-19. The results of this study partially revealed the working mechanism of XBJ in protecting cardiac function and controlling cytokine storm upon systemic infection. Our results suggested that XBJ might be a safe option for protecting cardiac function, controlling infection-induced cytokine storm, and preventing bacteria superinfection in severe COVID-19 patients.

### Future Directions

Clearing of dying/dead cells and extracellular matrix is a key function of cardiac resident macrophages (Li et al., 2021). Enhancing macrophage efferocytosis can activate the anti-inflammatory program and reduce the damages caused by apoptotic neutrophils (Greenlee-Wacker, 2016). Limited studies have been conducted on the impact of cardiac resident macrophages on sepsis-induced cardiac dysfunction and the influence of XBJ on cardiac resident macrophages. Liu et al. found XBJ (5 mg/mL) enhanced the survival and the expression of M2 markers in LPS-stimulated mouse peritoneal macrophages (Liu et al., 2015). It is important to understand the influence of XBJ and C0127s on the bone marrow derived macrophages and cardiac resident macrophages in sepsis. Several important questions remain to be answered. It will be important to understand the influences of bone marrow-derived macrophages and cardiac resident macrophages on the cardiac function in septic shock models. It is not clear how XBJ and C0127s influence the function of macrophages in the cardiac tissue of the CLP mice. Whether they influence the infiltration of bone marrow derived macrophages in the CLP model is not known either.

GDF3 and Sectm1a have been shown to influence cardiac function of septic mice and are potential therapeutic targets of sepsis-induced cardiac dysfunction (Li et al., 2020b; Mu et al., 2020; Wang et al., 2020). XBJ and C0127s may impact cardiac function of septic mice by influence these potential therapeutic targets. How XBJ and C0127s regulate these signaling remains to be unveiled.

## Summary

In summary, XBJ protects cardiac function partially by easing the cytokine storm during systemic infection. Paeoniflorin and Hydroxysafflor yellow A are key compounds in XBJ which exert major functions of XBJ in managing septic shock. Like XBJ, they protected cardiac function in septic shock. NF-κB, TNF-α, and CXCL2 signaling are potential targets of XBJ and C0127s in infection-induced cardiac dysfunction. This work indicated XBJ may attenuate cytokine storm to protect the cardiac function of septic shock and COVID-19 patients.

## Data Availability

All datasets generated for this study are included in the article and the Supplementary data.
