# Supplementary material for: Paeoniflorin and Hydroxysafflor Yellow A in Xuebijing Injection Attenuate Sepsis-Induced Cardiac Dysfunction and Inhibit Proinflammatory Cytokine Production
Source: Front Pharmacol. 2021 Apr 13;11:614024. doi: 10.3389/fphar.2020.614024 (PMC8112230; doi:10.3389/fphar.2020.614024)
Supplement: Supplementary file 1 [file Image1.tif]

Frontiers | Paeoniflorin and hydroxysafflor yellow A in Xuebijing injection attenuate sepsis-induced cardiac dysfunction and inhibit proinflammatory cytokine production | Pharmacology


- About
- Journals
- Research Topics
- Articles
- More

Submit

My Frontiers

Office

- TSOF
  - TSOF
  - Article Production

Typesetter 3

frontiersproduction@tnq.co.in

- Profile
- Settings & Privacy
- Help Center
- Logout

Submit

**Impact Factor 4.225** | **CiteScore 5.0**More on impact ›

|  |  |
| --- | --- |
| Frontiers in Pharmacology | Experimental Pharmacology and Drug Discovery |

Toggle navigation


Section


- (current)Section
- About
- Articles
- Research topics
- For authors 
  - Why submit?
  - Fees
  - Article types
  - Author guidelines
  - Review guidelines
  - Submission checklist
  - Contact editorial office
  - Submit your manuscript
- Editorial board

- *Article alerts*

##### This article is part of the Research Topic

Sepsis: Basic, Clinical and Therapeutic Approaches
View all
3
Articles

Articles


**Suggest a Research Topic >**

- 111
  total views

 View Article Impact

**Suggest a Research Topic >**

##### SHARE ON

- Facebook

  0
- Twitter

  0
- LinkedIn

  0
- AddThis

  New


## Original Research ARTICLE

Front. Pharmacol.
| doi: 10.3389/fphar.2020.614024

# Paeoniflorin and hydroxysafflor yellow A in Xuebijing injection attenuate sepsis-induced cardiac dysfunction and inhibit proinflammatory cytokine production Provisionally accepted The final, formatted version of the article will be published soon. **Notify me**

Xin-Tong Wang1, Zhen Peng1, 
Ying-Ying An1, 
Ting Shang1, 
 Guangxu Xiao1,  Shuang He1,  Xi Chen1, Han Zhang1, 
 Yuefei Wang1, Tao Wang1, 
Jun-Hua Zhang1, 
 Xiumei Gao1,  Yan Zhu1 and  Yuxin Feng1\*

- 1Tianjin University of Traditional Chinese Medicine, China

Sepsis-induced myocardial dysfunction is a major contributor to the poor outcomes of septic shock.   
As an add-on with conventional sepsis management for over 15 years, the effect of Xuebijing injection (XBJ) on the sepsis-induced myocardial dysfunction was not well understood. The material basis of Xuebijing injection (XBJ) in managing infections and infection-related complications remains to be defined. A murine cecal ligation and puncture (CLP) model and cardiomyocytes in vitro culture were adopted to study the influence of XBJ on the infection-induced cardiac dysfunction. XBJ significantly improved the survival of septic-mice and rescued cardiac dysfunction in vivo. RNA-seq revealed XBJ attenuated the expression of proinflammatory cytokines and related signalings in the heart which was further confirmed on the mRNA and protein levels. Xuebijing also protected cardiomyocytes from LPS-induced mitochondrial calcium ion overload and reduced the LPS-induced ROS production in cardiomyocytes. The therapeutic effect of XBJ was mediated by the combination of paeoniflorin and hydroxysafflor yellow A (HSYA) (C0127-2). C0127-2 improved survival of septic mice, protected their cardiac function and cardiomyocytes while balancing gene expression in cytokine-storm related signalings, such as TNF-α and NF-κB. In summary, Paeoniflorin and HSYA are key active compounds in XBJ for managing sepsis, protecting cardiac function, and controlling inflammation in the cardiac tissue partially by limiting the production of IL-6, IL-1β, and CXCL2.

Keywords: 
Xuebijing injection, septic shock, Myocardial depression, Paeoniflorin (Pae), Hydroxysafflor yellow A (HSYA), Cytokine storm, CXCL2/MIP-2

Received: 05 Oct 2020;
Accepted: 07 Dec 2020.

Copyright: © 2020 Wang, Peng, An, Shang, Xiao, He, Chen, Zhang, Wang, Wang, Zhang, Gao, Zhu and Feng. This is an open-access article distributed under the terms of the Creative Commons Attribution License (CC BY). The use, distribution or reproduction in other forums is permitted, provided the original author(s) and the copyright owner(s) are credited and that the original publication in this journal is cited, in accordance with accepted academic practice. No use, distribution or reproduction is permitted which does not comply with these terms.

\* Correspondence: 
Prof. Yuxin Feng, Tianjin University of Traditional Chinese Medicine, Tianjin, China, fengyn@live.com

Write a comment...

Add

##### COMMENTARY

##### ORIGINAL ARTICLE

##### People also looked at

## Enhancing Extracellular Adenosine Levels Restores Barrier Function in Acute Lung Injury Through Expression of Focal Adhesion Proteins

Wei Wang, Ning-yuan Chen, Dewei Ren, Jonathan Davies, Kemly Philip, Holger K. Eltzschig, Michael R. Blackburn, Bindu Akkanti, Harry Karmouty-Quintana and Tingting Weng

## Emodin Alleviates Severe Acute Pancreatitis-Associated Acute Lung Injury by Inhibiting the Cold-Inducible RNA-Binding Protein (CIRP)-Mediated Activation of the NLRP3/IL-1β/CXCL1 Signaling

Hailong Chen, Qiushi Xu, Mengfei Wang, Haoya Guo, Huanhuan Liu, Guixin Zhang and Caiming Xu

## Potential of Immune-Related Therapy in COVID-19

Chunjue Yuan, Ruoyun Li, Guohong Liu and Yunbao Pan

## Pharmacological Modulation of BET Family in Sepsis

Nian Wang, Runliu Wu, Paul B. Comish, Rui Kang and Daolin Tang

**Suggest a Research Topic >**

×

#### Supplementary Material

  

There is no supplementary material currently available for this article

Loading supplemental data...

  

|  | File Name |  |
| --- | --- | --- |
|  | Table 1.XLS |  |
|  | Table 2.XLS |  |
|  | Table 3.XLS |  |
|  | Table 4.XLS |  |
|  | Image 1.TIF |  |
|  | Image 2.tif |  |
|  | Image 3.TIF |  |
|  | Image 4.TIF |  |
|  | Image 5.TIF |  |
|  | Image 6.TIF |  |
|  | Image 7.TIF |  |
|  | Image 8.TIF |  |

  

Close

- About Frontiers
- Institutional Membership
- Books
- News
- Frontiers' social media
- Contact
- Careers
- Submit
- Newsletter
- Help Center
- Terms & Conditions
- Privacy Policy

© 2007 - 2021 Frontiers Media S.A. All Rights Reserved

### Privacy Preference Center

Our website uses cookies that are necessary for its operation. Additional cookies are only used with your consent. These cookies are used to store and access information such as the characteristics of your device as well as certain personal data (IP address, navigation usage, geolocation data) and we process them to analyse the traffic on our website in order to provide you a better user experience, evaluate the efficiency of our communications and to personalise content to your interests. Some cookies are placed by third-party companies with which we work to deliver relevant ads on social media and the internet. Click on the different categories' headings to change your cookie preferences. Click on "More Information" if you wish to learn more about how data is collected and shared.
More information

### Manage Consent Preferences

#### Strictly Necessary Cookies

Always Active

These cookies are necessary for the website to function and cannot be switched off in our systems. They are usually only set in response to actions made by you which amount to a request for services, such as setting your privacy preferences, logging in or filling in forms. You can set your browser to block or alert you about these cookies, but some parts of the site will not then work. These cookies do not store any personally identifiable information.

#### Analytics Cookies

Analytics Cookies

These cookies allow us to count visits and traffic sources so we can measure and improve the performance of our site. They help us analyse which pages are the most and least popular and see how visitors move around the site.    All information these cookies collect is aggregated and therefore anonymous.

#### Functional Cookies

Functional Cookies

These cookies enable the website to provide enhanced functionality and personalisation. They may be set by us or by third party providers whose services we have added to our pages. If you do not allow these cookies then some or all of these services may not function properly.

#### Advertising Cookies

Advertising Cookies

These cookies may be set through our site by our advertising partners. They may be used by those companies to build a profile of your interests and show you relevant adverts on other sites.    They do not store directly personal information, but are based on uniquely identifying your browser and internet device. If you do not allow these cookies, you will experience less targeted advertising.

### Back Button Performance Cookies

Vendor Search  Search Icon

Filter Icon

Clear

checkbox label label

Apply Cancel

Consent Leg.Interest

checkbox label label

checkbox label label

checkbox label label

Confirm My Choices
